# Assessing the sensitivity of the polio environmental surveillance system

**DOI:** 10.1371/journal.pone.0208336

**Published:** 2018-12-28

**Authors:** Steve J. Kroiss, Maiwand Ahmadzai, Jamal Ahmed, Muhammad Masroor Alam, Guillaume Chabot-Couture, Michael Famulare, Abdirahman Mahamud, Kevin A. McCarthy, Laina D. Mercer, Salman Muhammad, Rana M. Safdar, Salmaan Sharif, Shahzad Shaukat, Hemant Shukla, Hil Lyons

**Affiliations:** 1 Institute for Disease Modeling, Bellevue, WA, United States of America; 2 National Emergency Operations Centre for Polio Eradication, Kabul, Afghanistan; 3 World Health Organization, Geneva, Switzerland; 4 Department of Virology, National Institute of Health, Chak Shahzad, Islamabad, Pakistan; 5 World Health Organization, Islamabad, Pakistan; 6 National Emergency Operations Centre for Polio Eradication, Islamabad, Pakistan; University of Liverpool, UNITED KINGDOM

## Abstract

**Background:**

The polio environmental surveillance (ES) system has been an incredible tool for advancing polio eradication efforts because of its ability to highlight the spatial and temporal extent of poliovirus circulation. While ES often outperforms, or is more sensitive than AFP surveillance, the sensitivity of the ES system has not been well characterized. Fundamental uncertainty of ES site sensitivity makes it difficult to interpret results from ES, particularly negative results.

**Methods and findings:**

To study ES sensitivity, we used data from Afghanistan and Pakistan to examine the probability that each ES site detected the Sabin 1, 2, or 3 components of the oral polio vaccine (OPV) as a function of virus prevalence within the same district (estimated from AFP data). Accounting for virus prevalence is essential for estimating site sensitivity because Sabin detection rates should vary with prevalence—high immediately after supplemental immunization activities (SIAs), but low in subsequent months. We found that most ES sites in Pakistan and Afghanistan are highly sensitive for detecting poliovirus relative to AFP surveillance in the same districts. For example, even when Sabin poliovirus is at low prevalence of ~0.5–3% in AFP surveillance, most ES sites have ~34–50% probability of detecting Sabin. However, there was considerable variation in ES site sensitivity and we flagged several sites for re-evaluation based on low sensitivity rankings and low wild polio virus detection rates. In these areas, adding new sites or modifying collection methods in current sites could improve sensitivity of environmental surveillance.

**Conclusions:**

Relating ES detections to virus prevalence significantly improved our ability to evaluate site sensitivity compared to evaluations based solely on ES detection rates. To extend our approach to new sites and regions, we provide a preliminary framework for relating ES and AFP detection rates, and descriptions of how detection rates might relate to SIAs and natural seasonality.

## Introduction

Environmental surveillance (ES) for the detection of poliovirus circulation in urban areas has increased in scope and importance for global polio eradication efforts over the past decade [1], especially in countries recently or currently at the forefront of eradication efforts, such as Afghanistan, Pakistan, and Nigeria. ES has been particularly useful for complementing acute flaccid paralysis (AFP) case surveillance to provide awareness of the spatial and temporal extent of poliovirus circulation. In turn, the increase in evidence of transmission has led to more demand for ES sampling locations [1,2]. ES has been shown to be more sensitive than AFP surveillance [1–7], but the overall sensitivity of the ES system has not been well characterized, and there are many contributing factors [1,8,9]. This makes it challenging to interpret results from ES, particularly for negative results.

Evaluating the sensitivity of the ES system has several applications, both operational and programmatic. Operationally, such methods may be used to evaluate the relative sensitivity of newly created ES sites and as a diagnostic for sites that have low detection rates. The quantitative framework itself may be useful for identifying site-level factors associated with sensitivity. Programmatically, we can use this analysis to help inform the probability that wild poliovirus (WPV) or vaccine-derived poliovirus (VDPV) is truly absent from areas with negative ES detections. It is also relevant for monitoring oral polio vaccine (OPV) cessation for prolonged transmission of Sabin-like viruses.

In part, the increased sensitivity of ES compared to AFP is due to the low case to infection ratio for poliomyelitis: in a fully susceptible population, approximately 1 in 200 infections for type 1 (WPV1) or 1 in 2000 for type 2 VDPV infections exhibit paralytic symptoms [10–12]. Regardless of presenting symptoms, all infected individuals shed large amounts of poliovirus in their feces for several weeks which can be detected for days to months in sewage contaminated waste water [5]. Moreover, converging sewage networks can potentially monitor large at-risk populations.

ES sampling locations do not share the same physical characteristics nor the same epidemiological relevance. Site characteristics, like sewage flow, drainage area, and the presence of industrial wastes can help in selecting areas most likely to contain the polioviruses that are circulating within a community [13], but ongoing monitoring is required to build up evidence of site performance and validate its quality. In areas where wild or vaccine-derived polioviruses are circulating, it can be sufficient to detect these viruses to validate a specific ES site’s usefulness. In the absence of these viruses, the detection of Sabin strains from OPV use and non-polio enteroviruses (NPEV) may be used as proxy indicators to regularly monitor, validate, and compare site sensitivity.

Multiple methods can be used to evaluate ES site sensitivity. One approach is to use raw detection rates of virus (e.g., 3/12 positive samples per year) as a way to evaluate site sensitivity. However, this approach does not account for virus prevalence (the proportion of the population infected) in the surrounding population. For example, we would expect Sabin detection rates in ES to be high immediately after mass immunization campaigns, referred to as supplementary immunization activities (SIAs), but low in subsequent months [14]. This is particularly relevant in polio endemic countries in which there are several SIAs per year.

Isolation of Sabin-like viruses in sewage after mass immunization has a long documented history, dating to pre-licensure use of OPV [15–17]. In 1962, Riordan [15] documented administrative records of vaccine recipients in the respective catchments of sewage sampling sites. Recently, Berchenko et al [18] similarly paired administrative records of OPV vaccination in well-defined sewage site catchments with laboratory-quantified virus to study the ability to estimate infection prevalence from virus quantification in the laboratory. Several studies have compared Sabin isolation in stool sampling of a study population with sewage sampling (for example, [19,20]) in the context of mass immunization. Lodder et al [21] compare differential shedding and subsequent detection in sewage of an elderly population with different immunization histories. Blake et al [22] studied rates of Sabin type 2 detection in environmental surveillance sites related to OPV2 use in vaccination campaigns for the purposes of evaluating the global switch from trivalent OPV to bivalent OPV in 2016.

We study an approach to characterizing ES site sensitivity in Pakistan and Afghanistan relating the ES detection rate to the underlying virus prevalence in the surrounding population, as measured by the virus isolation rate in non-polio cases of AFP (NP-AFP). We focus on modeling Sabin-like viruses (SL1, SL2, SL3) and NPEV because these viruses are generally much more prevalent than WPV or VDPV and because this enables sensitivity assessment in areas without circulation of WPV or VDPV. NP-AFP data are continuously collected as part of routine surveillance and inform on virus prevalence in the underlying community without additional recruitment of a study population. Considering the probability of detecting any specific virus (WPV, SL, and/or NPEV) is affected by the testing algorithm established by the Global Polio Laboratory Network [23], we adjust for the implications of the laboratory procedures. We also model how the prevalence of these viruses changes over time, in response to seasons and SIAs. Then, we align the ES sample collection dates with daily estimates of virus prevalence, and thus relate the ES detection rate to virus prevalence to measure ES site sensitivity. Finally, we provide recommendations for extending our approach to evaluate new sites or regions without extensive modeling requirements.

## Methods

### Data

Sabin-like and NPEV detection rates were based on NP-AFP case surveillance data and environmental surveillance (ES) data from 2009–2016 in Pakistan and Afghanistan. We obtained AFP and ES data from the National Emergency Operations Centres for Polio Eradication in Afghanistan and Pakistan. We limited our analyses to data collected prior to OPV2 cessation (April 30, 2016) because of the substantial shifts in SL2 prevalence [14]. We also eliminated any ES sites from our analyses that were new in 2016 because of limited sample sizes. Institutional ethics approval was not sought for AFP surveillance data as they are retrospective and anonymized.

AFP surveillance consists of case reports from health facilities and active community searches for children with symptoms compatible with polio. When AFP cases are reported, two stool samples are collected within 14 days of each other and tested for polioviruses [23]. In most instances, the cause of paralysis is not polioviruses (NP-AFP). Since lab testing also flags the presence of Sabin strains in stool, we used NP-AFP cases to measure Sabin prevalence among the sampled population. Specifically, we used AFP case surveillance data that was not positive for WPV or VDPV. We assumed that NP-AFP cases with Sabin virus were not vaccine-associated paralytic polio (VAPP) cases, which could inflate our prevalence estimates. VAPP is exceedingly rare (~1 case per 900,000 first doses of OPV) and thus unlikely to significantly influence our results [24]. Our dataset included a total of 10,868 and 31,287 NP-AFP cases from Afghanistan and Pakistan respectively from 2009–2016.

Environmental surveillance consists of monthly wastewater grab samples collected from areas with a history of wild poliovirus cases, inadequate immunization coverage, and perceived risk of importation of wild poliovirus [25–27]. From 2009–2016, environmental surveillance was conducted at 14 sites in Afghanistan and 37 sites in Pakistan with sites being established at different times. This dataset included a total of 304 and 1,967 ES samples from Afghanistan and Pakistan respectively from 2009–2016. Additional details may be found in section A in S1 Text.

Virus isolation was done for all AFP and ES samples according to the protocols recommended by the World Heal Organization [25]. Briefly, samples were concentrated, extracted with chloroform, and inoculated into two different cell culture lines—L20B cells and RD cells [25]. AFP samples were inoculated into one flask of each cell line while ES samples were inoculated onto five L20B flasks and one RD flask to increase sensitivity. The L20B cells are genetically engineered mouse L-cells expressing poliovirus receptors and are highly selective for polioviruses [28]. The RD cells are human rhabdomyosarcoma cells that are susceptible to several type of enteroviruses, including polio [29]. RD cell isolates were cross-passaged on L20B cells to isolate potential polioviruses. If polioviruses were isolated from cross-passaged isolates, polioviruses were reported in the RD flask and no other viruses were reported (censoring of potential NPEV results). If enteroviruses were detected in the RD cells, but are not identified as polioviruses, they were classified as non-polio enteroviruses (NPEV) and were not further classified.

Poliovirus type differentiation was done for all culture isolates showing viral cytopathic effects using real-time reverse-transcription PCR (rRT-PCR) according to WHO recommendations [30–32]. Isolates were either classified as wild poliovirus, Sabin-like poliovirus, or vaccine-derived poliovirus. Isolates was classified as SL (Sabin-like) if still similar to the parent Sabin strain, having not passed a type-specific threshold of genetic divergence indicating classification as a VDPV [24].

### Modeling approach

**Fig 1:**
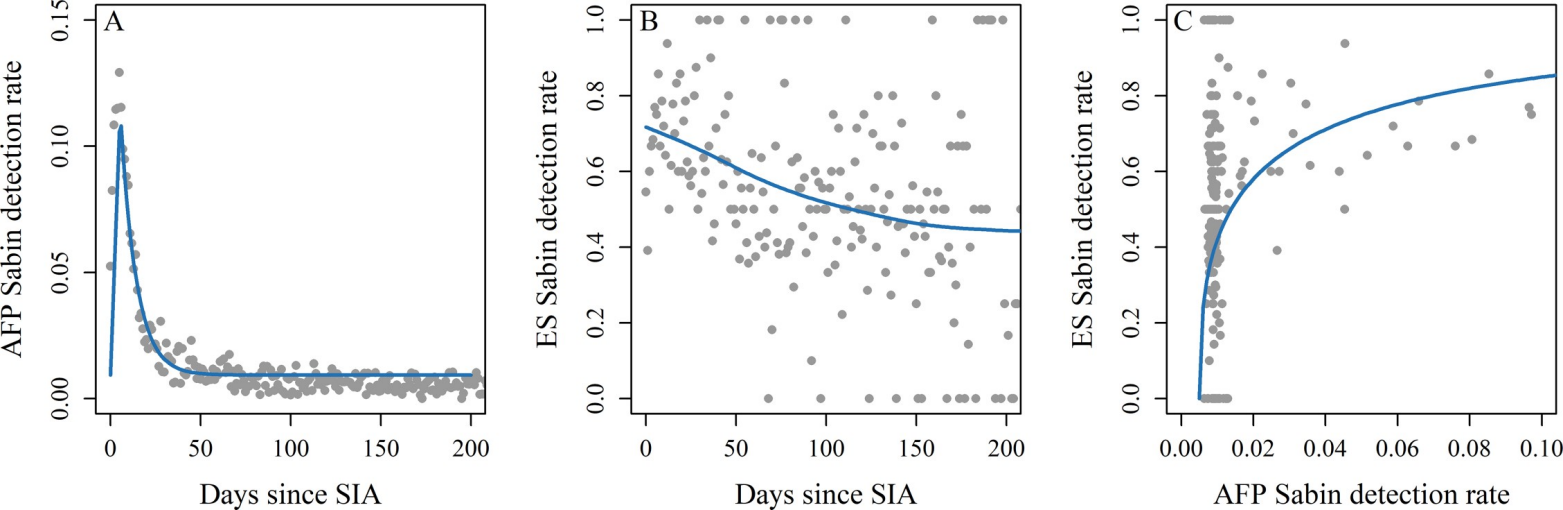
Sabin-like type 2 (SL2) detection rates in AFP and ES after mass supplemental immunization activities (SIA) in Pakistan. Panel A and B illustrate how the raw detection rates of SL2 in AFP and ES decline after OPV2 use in an SIA (either mOPV2 or tOPV). The ES data exhibit much more noise than AFP, partly due to the smaller sample size of ES and to the noisier nature of ES data. Panel C shows that raw detection rates of SL2 in ES increase as SL2 prevalence increases (prevalence estimates from model built in panel A). The grey dots are data from across all of Pakistan and the blue lines indicate mean model estimates.

To estimate the sensitivity of each ES site for detecting polioviruses, we took a two-stage approach. First, we used NP-AFP data to estimate the typical prevalence of SL1, SL2, SL3, and NPEV within the district and on the collection date of each ES sample (Fig 1A). Second, we examined the probability that each ES sample was positive for SL1, SL2, SL3, or NPEV based on the prevalence of each virus in the surrounding district (Fig 1C). We modeled the sensitivity to NPEV separately from the Sabin viruses to account for different treatments of these viruses in the lab procedure. We describe these methods in detail below.

### SL prevalence

We estimated the prevalence of SL1, SL2, and SL3 on the collection date of each ES sample as a function of the time since the last SIA (Fig 1A; section B in S1 Text for full model details). We estimated prevalence at the district level in Pakistan and province level in Afghanistan. We discuss the limitations and implications of this approach in the Discussion. We also examined delayed peak detection rates in ES relative to AFP for Sabin after SIAs since ES may capture secondary transmission in populations typically not sampled by AFP (children >5 years old). We determined the amount of the delay between ES and AFP by comparing models fit to different time delays with likelihood ratio tests. Full details can be found in section C in S1 Text.

### NPEV prevalence

We estimated the prevalence of NPEV on the collection date of each ES sample with a seasonal model based on NP-AFP detection rates (see section D in S1 Text for full model details). NPEV prevalence displays strong seasonal patterns–generally peaking in prevalence from April until October with a slight decline in June (see Fig B in S1 Text). To reflect these seasonal patterns, we modeled NPEV prevalence at the one month time scale based on NP-AFP detection rates. We estimated prevalence at the district level in Pakistan and province level in Afghanistan.

### ES site sensitivity model for SL1, SL2, and SL3

To examine the sensitivity of each ES site to detecting polio, we jointly modeled the probability of each ES site detecting SL1, SL2, or SL3 in each sample as a function of virus prevalence. We accounted for correlations in detection rates among the virus types within each ES site (i.e., a good site for SL1 should also be a good site for SL2 and SL3). We also accounted for sample level correlations among the virus types (i.e., SL1 and SL3 likely to be co-detected because of bivalent OPV use). We used the log of the virus prevalence estimate from prevalence models fit to NP-AFP data as a correlate of ES detection probability [14].

Formally, we used a binomial regression with complementary log-log (cloglog) link to estimate the components of the detection probability *p*_*ijk*_ indexed by virus type *i*, ES site *j*, and sample k, that is

$$\begin{array}{c} y_{ijk}∼Bern(p_{ijk})\\ p_{ijk}=1-exp(-exp(\beta_{0i}+\beta_{1i}*\log\left(x_{ijk}\right)+u_{j}+v_{k})) \end{array}$$

Here, *y*_*ijk*_ represents positive (y = 1) or negative (y = 0) detections of virus, *β*_0*i*_ is the virus type specific intercept, *β*_1*i*_ is a virus type specific coefficients for virus prevalence indicator, *u*_*j*_ is a multivariate normal random error to account for correlations in ES site level detection rates among the virus types, and *v*_*k*_ is a multivariate normal random error to account for correlations in sample level detection rates among the virus types. We estimated model parameters using a Bayesian approach, with posterior samples generated through the Integrated Nested Laplace Approximation using the INLA package v0.0–1468872408 [33,34] in R v3.4.1 [35]; further details including prior distributions may be found in section E in S1 Text.

We performed model comparison to determine the impact of including virus prevalence in our models by using a likelihood ratio test to compare models with and without virus prevalence.

### ES site sensitivity model for NPEV

We modeled ES sensitivity to NPEV separately from Sabin because of the differential treatment of these virus types in the lab procedure. Specifically, the lab procedure censors NPEV results if polioviruses are detected in the RD flask. Since we did not have access to long-term flask level data (except for 2017; see below), we could not determine which samples detected polioviruses in the RD flask and thus censored NPEV results. As such, we corrected for potential censoring by only using NPEV results for samples without poliovirus detections (a relatively rare occurrence in our dataset). We modeled ES site sensitivity to NPEV in a similar fashion to the Sabin model; further details may be found in section E in S1 Text.

### Flask-level interference analysis

While our analysis accounts for sample level correlations in detection among SL1, SL2, and SL3, other polio viruses such as wild strains or VDPVs may interfere with detection. For example, it has been suspected that wild polioviruses may outcompete the Sabin viruses during the cell culture amplification step of the analysis. We explored this possibility with a permutation test on co-detection rates using flask level ES data for 2017 (see section G in S1 Text).

## Results

### Virus detection rates

**Fig 2:**
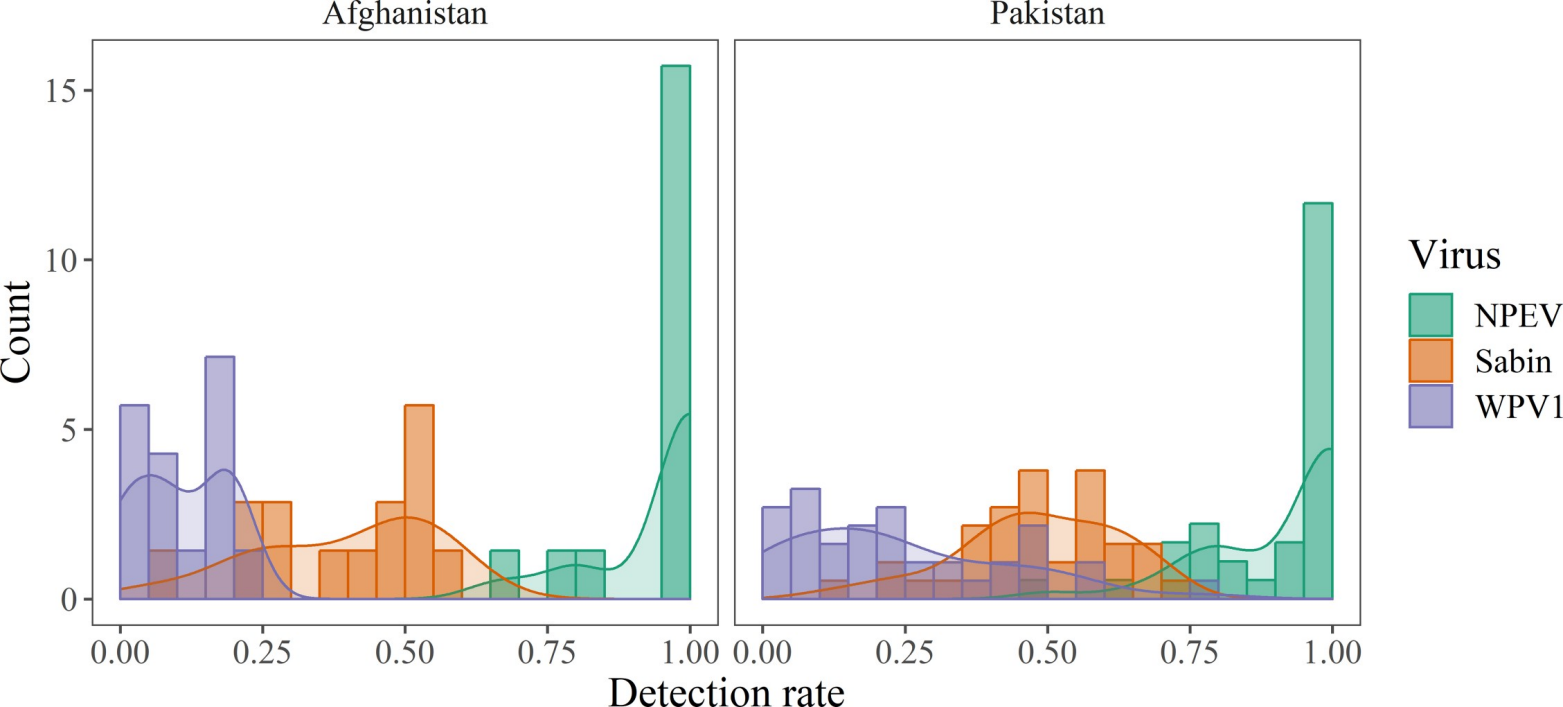
Histograms of ES sites listed by their detection rate of NPEV, Sabin, and WPV1 for Afghanistan and Pakistan. The bars indicate the proportion of positive samples for each virus within each ES site (Sabin averaged across SL1, SL2, and SL3). Detection rates for NPEV are based only on samples without other poliovirus detections to avoid the censoring effects of polioviruses on NPEV reporting. The lines indicate kernel density estimates for the corresponding data.

Virus detection rates were highly variable among ES sites, but detection rates were generally lowest for WPV; moderate for SL1, SL2, and SL3; and highest for NPEV (Fig 2, Table D in S1 Text). Approximately 30% of ES sites in Pakistan had WPV detection rates ≥33%, while all the sites in Afghanistan were <25%. In contrast, Sabin detection rates (averaged across SL1, SL2, and SL3) were ≥33% in 87% of sites in Pakistan and 65% of sites in Afghanistan. There were few ES samples to assess NPEV detection rates because poliovirus detection in the RD flask censors the reporting of NPEV. This limited us to using samples without any polioviruses, a relatively rare occurrence in this region, resulting in a median of 4 and 6 samples per site respectively in Afghanistan and Pakistan. Within these samples NPEV detection rates were 100% in 78% of sites in Afghanistan and 54% of sites in Pakistan. All of the sites detected NPEV in at least 50% of the samples.

### ES sensitivity analysis

**Fig 3:**
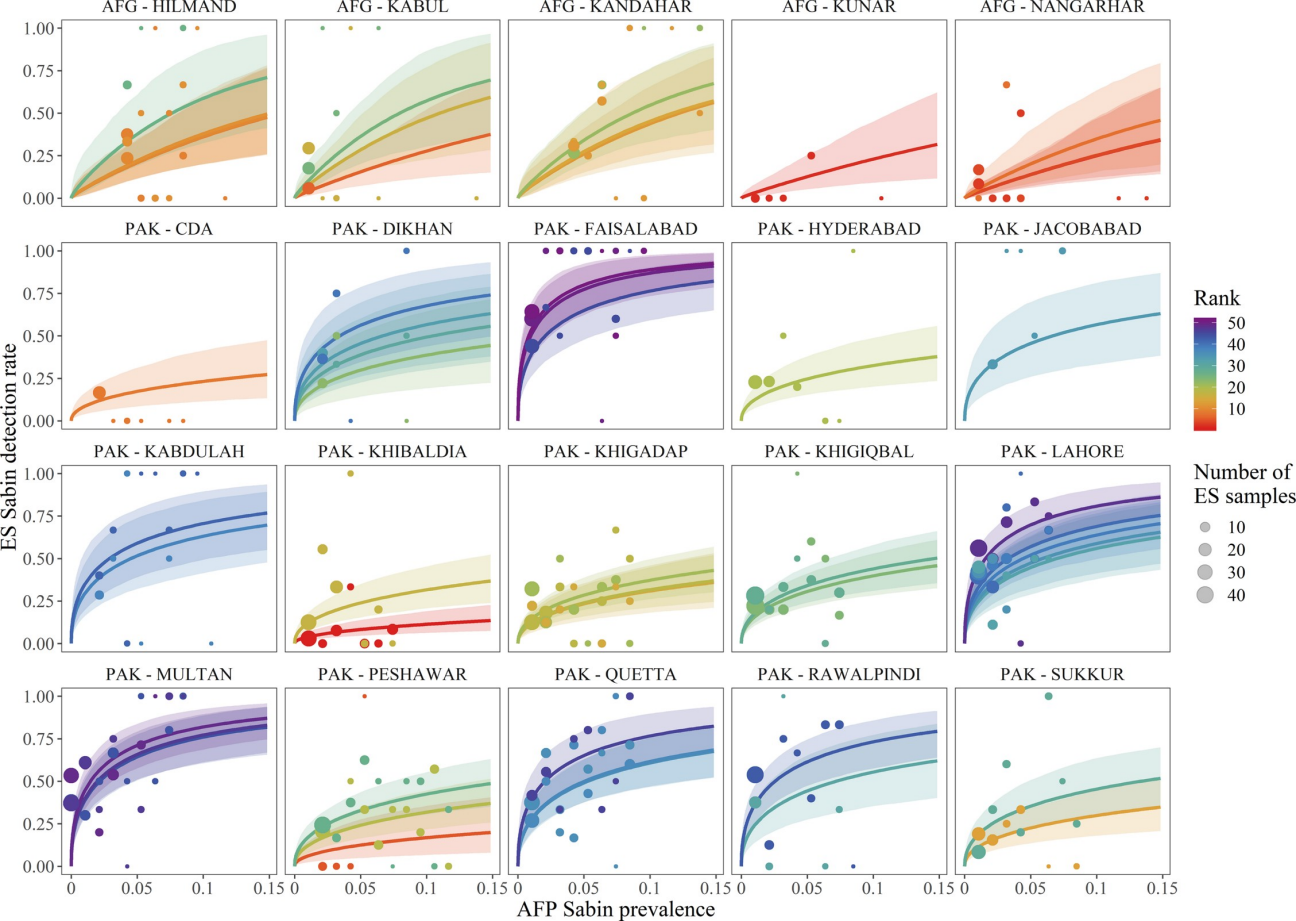
ES sensitivity curves for SL1 for each ES site in Pakistan. ES detection rates (sensitivity) of SL1 increase rapidly with increasing virus prevalence. The dots represent observed rates of Sabin detection from ES data and prevalence estimates based on AFP modeling. The dots are scaled to the number of samples. The solid lines represent mean model predictions for each ES site and the ribbons represent 95% credible intervals. Data and model estimates are colored by ES site from least sensitive (red) to most sensitive (purple). Sites were ranked based on their detection rates at the median predicted Sabin prevalence (0.03). Facets group ES sites at the province level within Afghanistan (AFG) and district level in Pakistan (PAK). See Fig F-H in S1 Text for the corresponding plots for SL2, SL3, and NPEV.

Our models relating Sabin detections with prevalence estimates indicated that ES is generally highly sensitive to poliovirus detection, and more sensitive than AFP on a per sample basis (SL1 results in Fig 3; SL2 and SL3 results in Fig F-G in S1 Text). For example, >30 days after SIAs, the rate of detecting Sabin in a single NP-AFP case is ~0.5–3% (averaged across SL1, SL2, and SL3), but ~34–50% in a single ES sample. As expected, ES detection rates increased when Sabin prevalence was highest shortly after an SIA. For example, Sabin virus prevalence peaks around 3–10% in the first 2 weeks after an SIA [14] and ES detection rates during this period range from 44–67%.

We evaluated the impact of including virus prevalence in our Sabin models by comparing models with and without virus prevalence. Likelihood ratio tests indicated that the Sabin prevalence model was significantly improved over the reduced model (P < 0.001; marginal log likelihoods were -4838 and -5003 respectively), demonstrating that relating ES detection to virus prevalence in the surrounding population significantly improved our ability to predict ES detection rates and site sensitivity.

Our models relating NPEV detections with prevalence estimates also indicated a highly sensitive ES system (Fig H in S1 Text) since detection rates were at or near 100% in most sites.

### Variation in sensitivity among sites

**Fig 4:**
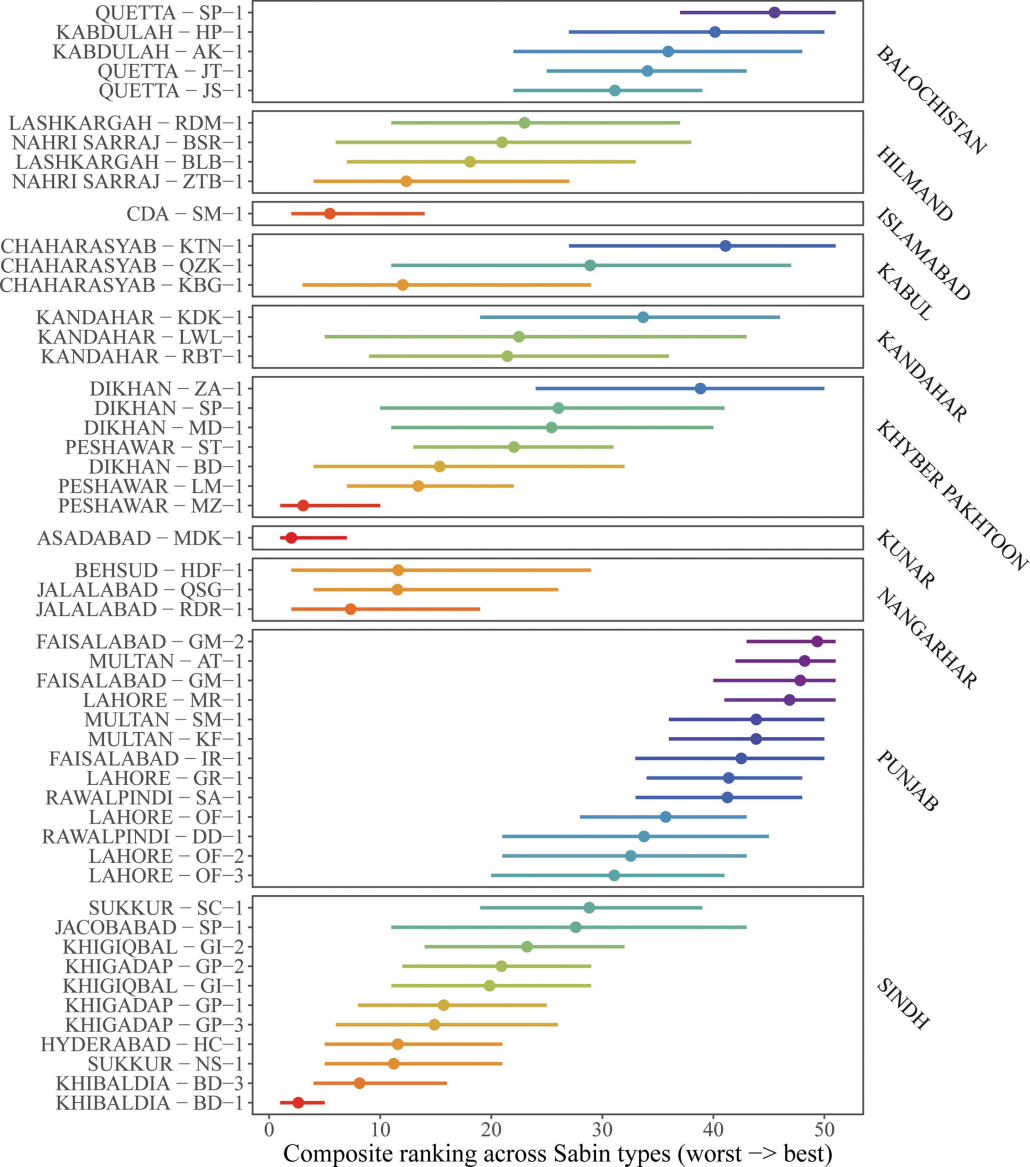
Composite model rankings of ES site sensitivity from least to most sensitive. We averaged rankings across SL1, SL2, and SL3 within each of the 500 model posteriors. Dots indicate the mean rank estimate and bars indicate 95% credible intervals. Sites are colored from least sensitive (red) to most sensitive (purple). ES sites are labeled with the district name and side ID (labels on the left) and grouped at the province level (labels on right).

Our analysis indicated substantial variation in Sabin sensitivity rates. The sites with the highest ranked Sabin sensitivity estimates occurred in Quetta, Faisalabad, Multan, Rawalpindi, and Lahore (Figs 3 & 4). All of these areas have ES sites with detection probabilities >75% at baseline Sabin prevalence. The lowest ranked sites occurred in Peshawar, Islamabad, Karachi, Kunar, Nangarhar, Sukkur, and Hyderabad and had detection probabilities <50% at baseline Sabin prevalence. Despite their low sensitivity estimates, these sites have still reported epidemiologically relevant data. For example, the ES site in Islamabad had several WPV detections in 2017. Unfortunately, the NPEV sensitivity analysis was too underpowered to discriminate sensitivity differences between sites (discussed in section F and Fig H in S1 Text).

### Flagging ES sites for evaluation

Our analysis of WPV detection rates and Sabin sensitivities lead us to flag several sites for investigation. We used a median split to flag the lower half of ES sites in terms of WPV detection rates which resulted in sites with WPV detection rates less than 11% and 20% in Afghanistan and Pakistan respectively. We further flagged sites using a median split on Sabin sensitivity rankings. Sites that were flagged for both low WPV detection rates and low Sabin sensitivity were considered the most in need of investigation–this included 3 sites in Afghanistan and 7 sites in Pakistan (Table D in S1 Text).

### Correlations in rankings

**Fig 5:**
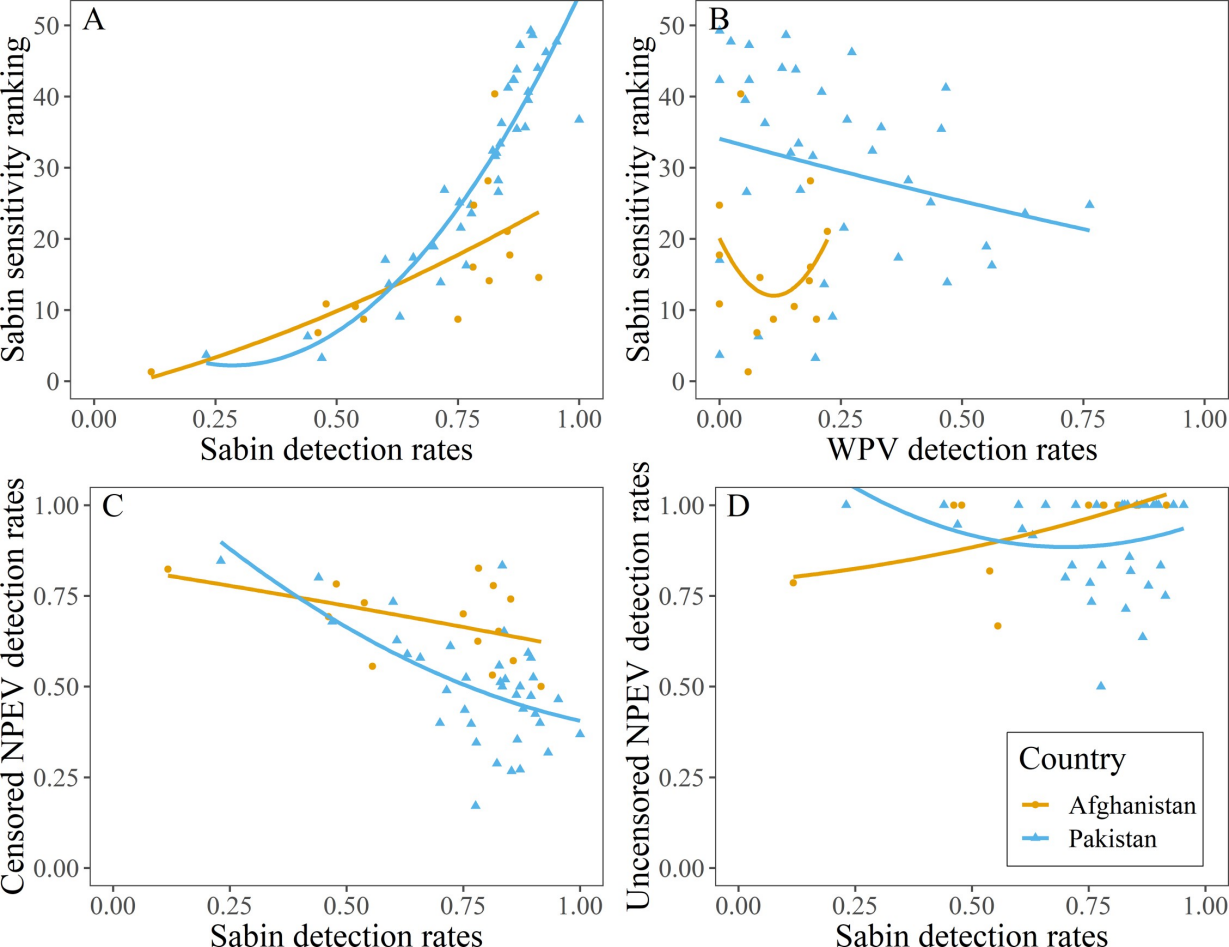
Correlations between model rankings and raw detection rates. Panel A illustrates the positive relationship between ES site sensitivity model rankings (averaged across SL1, SL2, and SL3) from low (small numbers) to high (high numbers) and detection rates of any type of Sabin at each site. Panel B shows the relationship between ES site sensitivity model rankings and WPV detection rates. Panel C shows the negative relationship between NPEV and Sabin detection rates across all samples, including those where polio was detected and could potentially censor NPEV reporting. Panel D shows the relationship between NPEV and Sabin detection rates when we correct for the potential censoring by only including NPEV results for samples without any poliovirus detections. The line represents a loess smoother fit to the data.

Mean ES site sensitivity model rankings (averaged across SL1, SL2, and SL3) were strongly positively correlated (Spearman’s rho = 0.74 and 0.93, 95% CI [0.34, 0.92] and [0.83, 0.96], for Afghanistan and Pakistan respectively) with raw detection rates of any kind of Sabin (Fig 5A) indicating that raw detection rates are a useful estimate of ES site sensitivity. However, there was scatter around this relationship suggesting that without accounting for virus prevalence, raw ES detection rates can give a biased interpretation of ES site sensitivity.

The composite model rankings of ES site sensitivity (averaged across SL1, SL2, and SL3) were slightly negatively correlated with raw detection rates of WPV in Pakistan (Spearman’s rho = -0.32, 95% CI [-0.61, 0.003]; Fig 5B), but we found no correlation in Afghanistan (Spearman’s rho = -0.02, 95% CI [-0.53, 0.60]). A negative correlation may result from a causal relationship—low vaccination rates would allow for low Sabin detection rates and a high potential for WPV circulation and detection, but this is not supported by examination of vaccination rates (section I in S1 Text). Alternatively, this could result from interference between WPV and Sabin in the isolation step, but this is not supported by the flask-level analysis (see below).

When we examined NPEV detection rates across all samples and ignored the potential censoring effects of polioviruses on NPEV results, we found a slight negative correlation between NPEV and Sabin detection rates in Pakistan (Fig 5C; Spearman’s rho = -0.46, 95% CI [-0.71, -0.14]), but no correlation in Afghanistan (Spearman’s rho = -0.43, 95% CI [-0.84, 0.11]). However, when we corrected for the effects of censoring by filtering the NPEV results to samples without any other polioviruses, we observed a positive relationship in Afghanistan (Fig 5D; Spearman’s rho = 0.53, 95% CI [0.17, 0.80]), but no relationship in Pakistan (and 0.08, 95% CI [-0.25, 0.37]). These results highlight the importance of understanding the lab algorithm when interpreting NPEV detection rates since poliovirus detection in the RD flask censors the reporting of NPEV.

### Delayed detections in ES relative to AFP

**Fig 6:**
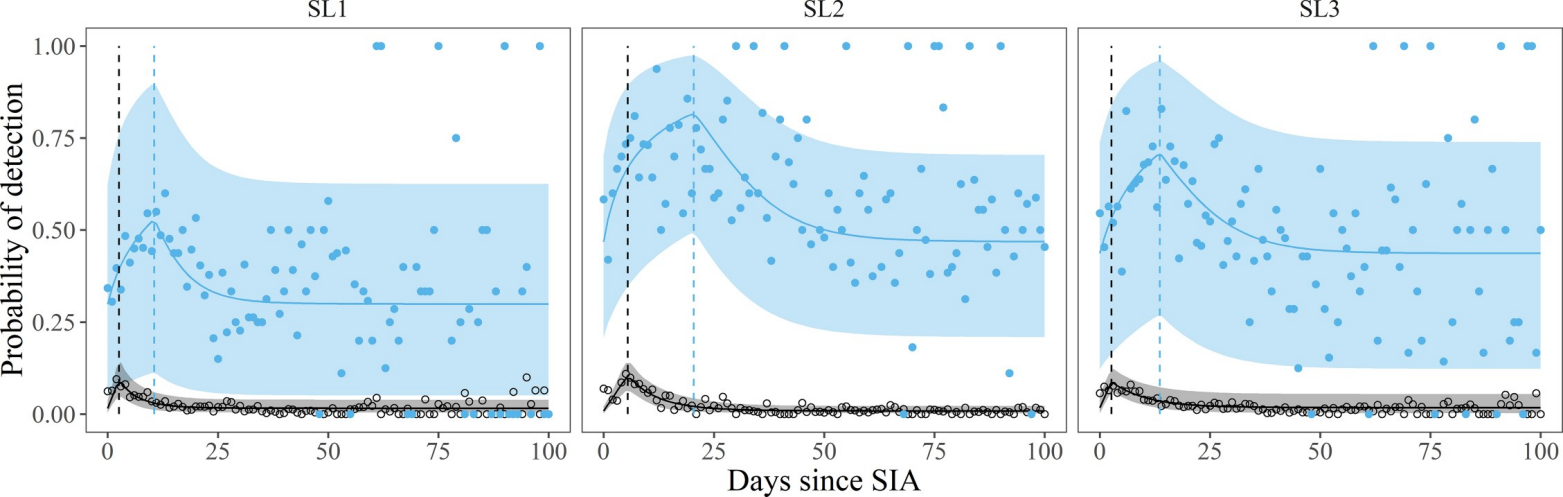
Peak ES detection of Sabin occurs later than in AFP after mass supplemental immunization activities (SIAs). The duration of the delay varied by type–with ES peaking later than AFP by 8, 15, and 11 days for SL1, SL2, and SL3 respectively. These delays suggest ES is able to detect significant amounts of secondary transmission after SIAs that is not typically captured in the AFP surveillance system. Figure elements are colored blue for ES and black for AFP. The dots represent observed detection rates across all of Pakistan. The solid lines represent mean model predictions and the ribbons represent 95% credible intervals across all of Pakistan. The vertical dashed lines indicate the timing of peak detection rates for each surveillance system. The panels group data by serotype: SL1, SL2, and SL3.

Model selection indicated that peak ES detection rates occurred 8, 15, and 11 days after peak SL1, SL2, and SL3 NP-AFP prevalence respectively following SIAs in Pakistan (Fig 6 and Fig D in S1 Text). We shifted our prevalence estimates accordingly (section C in S1 Text). These delays in peak ES detection rates are likely the result of ES capturing secondary transmission in populations typically not sampled by AFP (children >5 years old). While the time shifted models better fit the Pakistan data, they did not appreciably change site sensitivity rankings (Fig D in S1 Text). There was only weak support for delayed models for Afghanistan, so we retained the original unshifted prevalence models.

### Flask-level interference analysis

While interference between WPV and Sabin has been suspected, co-detections occur regularly at both the sample and flask level. Co-detections occurred in 23.8% of samples and 9.8% of flasks in which at least one poliovirus was detected. Correlation and permutation tests both indicate there is no discernable interference between WPV and Sabin detection at the sample or flask level (see section G in S1 Text).

## Discussion

### Environmental surveillance captures shifts in virus prevalence

Our models indicated that relating ES detections to virus prevalence in the surrounding population significantly improved our ability to predict ES detection rates and site sensitivity since ES detection rates varied strongly with virus prevalence (Figs 1 & 4). Alternative approaches for evaluating ES site sensitivity, such as ranking by raw Sabin detection rates, fail to account for the impact of SIAs on virus prevalence and may lead to inaccurate estimates of sensitivity. While we noted a high correlation between the composite sensitivity ranking and raw Sabin detection rates, in many instances the raw detection rates over- or underestimated site sensitivity. Regional differences in the number of SIAs or vaccination coverage rates can influence virus prevalence and thus the expected rate of ES detections. The timing of sample collection also can have a large impact on detection rates. For example, ES samples collected in the first two weeks after an SIA were ~30% more likely to detect Sabin than samples collected more than a month after SIAs (Fig 6).

Relating ES detections to virus prevalence also indicated that ES is a considerably more sensitive tool for detecting polio than AFP on a per sample basis (Fig 3). The high sensitivity of ES makes it an ideal tool for monitoring rare events such as VDPV emergences, WPV transmission, and Sabin transmission after OPV cessation. For example, other studies have demonstrated that the pulse in Sabin detection rates after an SIA is typically only detectable in AFP for approximately 50 days [14], but with the increased sensitivity of ES, Sabin should be detectable for several months after an SIA (Figs 1B and 6). In fact, several studies have shown that ES has been able to detect Sabin for several months after OPV use [3,20,36–38].

Spatial heterogeneity in AFP surveillance sensitivity may influence our model results. For example, low AFP sensitivity could result from poor access, stool inadequacy (collection >14 days after paralysis onset), cold chain failure, etc. In these instances, Sabin prevalence could appear artificially low and inflate our estimates of ES site sensitivity. While differences in AFP sensitivity can occur, it’s typically less common in urban settings where most ES sites are located. Analysis of surveillance rates suggest that all districts have sufficient surveillance rates (section I in S1 Text).

Our analysis suggests that the ES network can detect significant amounts of secondary transmission of Sabin after SIAs that is not typically captured in AFP surveillance. This was evidenced by the 8–15 day delays in peak Sabin detection rates in ES relative to AFP following SIAs (Fig 6). The discrepancy between these datasets likely results from how each system draws samples from the population. ES samples from large populations connected to sewer or drainage systems, while AFP primarily samples children under the age of five. Secondary transmission of Sabin in older cohorts, perhaps those with waning mucosal immunity [39], could therefore account for the delayed peak in ES. The type specific differences in delay length further support transmission being at the root of the issue. For example, time delays between AFP and ES were greatest for SL2, then SL3, then shortest for SL1. This ordering corresponds exactly with the duration of the peaks in prevalence among Sabin types following SIAs–longest for SL2, then SL3, and shortest for SL1 [14]. These difference could result from type specific mucosal immunity differences since increasing exposure to Sabin reduces the probability of take and lowers the duration of shedding [40]. Moreover, SL2 is known to be more transmissible than the other types [40] which could explain it having the longest peak in prevalence and longest time to peak ES detection. While there was support for delays between AFP and ES in both countries, they were only strongly supported in Pakistan (Fig D in S1 Text). This is perhaps unsurprising since Pakistan has considerably more data than Afghanistan.

Our models may allow for the reverse estimation of Sabin prevalence in the surrounding population from ES detection rates. This may be particularly useful for VDPV or WPV since these viruses are often detected in ES samples before or without AFP cases being detected. In these instances, ES detection rates may at least provide a useful bound on the number of infections and the likelihood of missing cases in the surrounding region. For example, the number of infections can be estimated based on the ES detection rate, the population size in the ES catchment, and the case to infection ratio of the specific strain. While this approach doesn’t allow for precise estimates, changes in the frequency of ES detections can at least suggests shifts in prevalence.

### Reconciling WPV & SL1, SL2, and SL3 detection rates

The negative relationship between Sabin sensitivity rankings and WPV detection rates in Pakistan (Fig 5B) seemingly poses a challenge for reconciling site sensitivity depending on the metric used. However, sensitivity and WPV detection rates need not be positively correlated because sensitivity does not imply epidemiological relevance. For example, incredibly sensitive sites may occur in well immunized populations that don’t experience much or any WPV circulation. Regardless, there are potential reasons for a negative relationship between Sabin sensitivity and WPV detection rates that are interesting to explore. Two possibilities include interference during sample analysis, or an effect of low vaccination rates.

Competition or interference has long been suspected during sample analysis, the idea being that wild strains might outcompete vaccine strains in the cell culturing step. However, our permutation analysis of flask-level data indicates there is no discernable interference between wild and vaccine strains (section F in S1 Text). Moreover, the primers used to detect Sabin are known to be highly targeted and sensitive to Sabin detection [41]. The effect of differential vaccination rates seems a more plausible explanation.

Poor vaccination rates within ES catchments would lead to low SL1 prevalence and the resulting low immunity could allow for WPV circulation. The critical point is that SL1 prevalence in the catchment may be lower than the surrounding district, making site sensitivity appear worse than if we were able to estimate Sabin prevalence at the catchment level instead of at the district level. While the scale of our prevalence estimates is a deficiency of our approach, this issue may allow us to flag ES catchments with poor vaccination rates. For example, the catchment regions of ES sites with high detection rates of WPV but low Sabin detection rates could be targeted during SIAs. An in depth analysis of vaccination rates and their impact on sensitivity and detection rates is beyond the scope of this paper and should be pursued in future endeavors. Preliminary analysis of the relationship between sensitivity rankings and vaccination rates did not reveal strong evidence of either (section I in S1 Text).

### Flagging low sensitivity sites

Our analysis flagged several ES sites in Afghanistan and Pakistan based on the sites having both low WPV detection rates and low Sabin sensitivity rankings (Table D in S1 Text). Our recommendations for flagged sites are twofold. First, negative virus detections from flagged sites should be interpreted cautiously since these sites may be more likely to miss polioviruses than detect them. Extra caution should be taken with these sites as the world approaches eradication and the importance of surveillance increases [1,2,42,43]. Second, the location and methods used at these sites should be evaluated. By evaluation, we do not necessarily recommend that sites be closed. In several cases, even these low sensitivity sites have provided epidemiologically important information such as WPV or VDPV transmission. Instead, we recommend scrutiny of the site location, site conditions, and methods used for sample collection to determine if refinements might improve sensitivity. For example, sample collection sites could be moved down stream, larger sample volumes could be collected, or nearby sites could be added to increase site sensitivity. Environmental factors are also likely to play a large role in site sensitivity such as population size in the catchment, water flow rates, water temperature, and the hour of sample collection [1]. Future studies should explore these factors and determine their association with site sensitivity.

### Extending our approach to new sites and other regions

As new ES sites are created and other regions seek to evaluate the sensitivity of their sites, rough rules of thumb are needed to make evaluation quick and feasible without extensive modeling. We provide four main recommendations to extend our approach to new sites and regions.

First, expectations for ES detection rates should be scaled to AFP detection rates to account for temporal and regional differences in virus prevalence and vaccination rates. As a rough rule of thumb, separating ES detection rates within 30 days of an SIA and after 30 days accounts for the short pulse in Sabin detection rates [14]. Within 30 days of SIAs, Sabin detection rates in Pakistan and Afghanistan ES were ~6–15 times that of AFP cases and carried additional information about the time course of shedding after campaigns. After more than 30 days of SIAs, Sabin detection rates in Pakistan and Afghanistan ES were ~16–68 times higher than that from AFP data. Holding other regions with new ES deployments to these same standards will be difficult since Pakistan and Afghanistan have had years to refine their protocols and methodology, but at the very least it indicates ES detection rates should be several factors higher than in AFP in all countries that use OPV with or without SIAs.

Second, while raw Sabin detection rates are a good proxy for ES site sensitivity, there is variation around this relationship. Sites with poor Sabin detection may be epidemiologically relevant as evidenced by sites in Pakistan that are good at WPV detection and poor for Sabin detection. Thus, we urge caution in relying solely upon Sabin detection rates for evaluation of ES sites.

Third, large numbers of samples are typically necessary to determine the sensitivity of new sites due to the binary nature of observation. This is problematic due to the natural programmatic urgency in identifying useful ES sites or alternatively discarding them. Suppose we were to compare the detection rate of one virus type in a new site to a benchmark detection rate to decide whether a site should be discontinued (both detection rates assumed constant); for illustration, suppose the benchmark detection rate is 0.47, the median detection rate for sites in Pakistan for SL1 (at 0.03 prevalence). It would require 14 samples to determine that the lowest ranked site in our analysis (0.14 ES detection rate) had a lower detection rate than the benchmark with Type 1 error of 0.05 and power of 0.8. Even an extremely poorly performing site with a detection rate of 0.05 would need at least 9 samples to determine that it had a lower detection rate than the median ranked site. A site with more modest under-performance, a detection rate of 0.3, would require 52 samples to distinguish it from the benchmark. Since most ES sites are sampled on a monthly basis, confident evaluation of new sites will take considerable time, possibly years. More frequent sampling or better use of the samples–e.g. evaluation of multiple pathogens, quantification, etc.–may be scientifically advisable and yet difficult to operationalize.

Fourth, a clear understanding of lab methodology is vital for interpreting NPEV detection rates. Due to the censoring of NPEV detection when polioviruses are detected in the RD flask, we recommend that NPEV results be ignored if polioviruses are detected in that sample. When we corrected for potential censoring, we estimated that the majority of ES sites had NPEV detection rates of 100% and never less than 50%. If flask level data are available, more NPEV results could be examined if polioviruses are absent from the RD flask. Other approaches have been suggested, such as assuming that NPEV would’ve been detected whenever polio is also detected, but we urge extreme caution with this approach. In Pakistan and Afghanistan, this approach would lead to nearly every site having 100% NPEV detection rates, thus providing very little new information for ranking. NPEV may have greater utility for ranking sites in regions with lower poliovirus detection rates.

## Conclusions

This study evaluates ES site sensitivity by comparing ES detection rates with evidence of community-level background infection prevalence of SL and NPEV obtained from AFP surveillance data. A full evaluation of the usefulness of an ES site should also include additional factors, for example site attributes and epidemiological relevance as indicated by past detection of WPV or cVDPV or otherwise at-risk populations.

While our approach has limitations, we believe that our analysis is part of the solution for better understanding and evaluating patterns of observations in ES systems in Pakistan, Afghanistan, and elsewhere. Our models also provide a preliminary framework for extending our approach to new sites and regions by relating ES and AFP detection rates, and understanding how they might relate to SIAs and natural seasonality. For example, new sites could be evaluated by separately grouping detections within 30 days of an SIA when Sabin should be easy to detect, and more than 30 days after an SIA when Sabin should be harder to detect. As the ES system has expanded to include at least 30 countries, evaluations of the sensitivity of these new sites will be needed to understand their utility.

Environmental surveillance will be increasingly useful as the world nears the eradication of polio However, ES cannot be universally deployed which necessitates continued vigilance for maintaining the AFP surveillance system. In fact, both ES and AFP surveillance will need to be maintained for several years after the last cases have been observed because polioviruses have been known to circulate silently for several years [44,45].

Environmental surveillance may also effectively monitor other pathogens besides poliovirus. For example, there is growing interest in developing an environmental surveillance network for typhoid since the WHO recently recommended introduction of the typhoid conjugate vaccine in countries with the highest burden of typhoid disease [46]. However, there is a lack of clinical typhoid reporting sites to aid vaccination decision-making [47,48]. An environmental surveillance network for typhoid would be a useful complement or less expensive alternative to clinical surveillance. Our work illustrates a means by which to benchmark and compare sites in an environmental surveillance network.

## Supporting information

S1 Text Supplemental information and analyses. Additional site information, model details, and results.
(DOCX)

## Abbreviations

AFP: acute flaccid paralysis surveillance
ES: environmental surveillance
L20B: a mouse L-cell culture line genetically engineered to express poliovirus receptors and be highly selective for polioviruses
mOPV2: monovalent oral polio vaccine containing only Sabin 2
NPEV: non-polio enterovirus
NP-AFP: non-polio acute flaccid paralysis
OPV: oral polio vaccine
RD cells: a human rhabdomyosarcoma cell culture line susceptible to several type of enteroviruses, including polio
RI: routine immunization
SIA: supplemental immunization activity
SL1, SL2, or SL3: Sabin-like type 1, 2, or 3 viruses respectively
tOPV: trivalent oral polio vaccine containing Sabin 1, 2, and 3
VDPV: vaccine-derived polioviruses
WPV: wild polio virus
