# Supplementary material for: Assessing the sensitivity of the polio environmental surveillance system
Source: PLoS One. 2018 Dec 28;13(12):e0208336. doi: 10.1371/journal.pone.0208336 (PMC6310268; doi:10.1371/journal.pone.0208336)
Supplement: S1 Text — Additional site information, model details, and results. (DOCX) [file pone.0208336.s001.docx]

**Supporting information**

**Assessing the sensitivity of the polio environmental surveillance system**

Steve J. Kroiss, Maiwand Ahmadzai, Jamal Ahmed, Muhmmad Masroor Alam, Guillaume Chabot-Couture, Michael Famulare, Abdirahman Mahamud, Kevin A. McCarthy, Laina Mercer, Salman Muhammad, Rana M. Safdar, Salmaan Sharif, Shahzad Shaukat, Hemant Shukla, and Hil Lyons

**Table of Contents**

[A Environmental surveillance sites 1](#_Toc528567997)

[B SL prevalence model 1](#_Toc528567998)

[C Delayed peak detection rate in ES relative to AFP 3](#_Toc528567999)

[D NPEV prevalence model 7](#_Toc528568000)

[E ES site sensitivity model sampling 8](#_Toc528568001)

[F ES site sensitivity results 9](#_Toc528568002)

[G Flask-level interference analysis 11](#_Toc528568003)

[H Site information and virus detection rates 15](#_Toc528568004)

[I Potential correlates of ES sensitivity rankings 17](#_Toc528568005)

[References 17](#_Toc528568006)

A Environmental surveillance sites

From 2009-2016, environmental surveillance was conducted at 14 sites in Afghanistan in the districts of Lashkargah (2 sites) and Nahri Sarraj (2) in Hilmand province; Chaharasyab district (3) in Kabul province; Kandahar district (3) in Kandahar province; Asadabad district (1) in Kunar province, and Behsud (1) and Jalalabad (2) district in Nangarhar province. Environmental surveillance was also collected at 37 sites in Pakistan in the same time period in the districts of Quetta (3 sites) and Killa Abdullah (2) in Balochistan province; Islamabad (1), the federal capital; Dera Ismail Khan (4) and Peshawar (3) district in Khyber Pakhtoon province; Faisalabad (3), Multan (3), Lahore (5), and Rawalpindi (2) district in Punjab province; and Sukkur (2), Jacobabad (1), Baldia (2), Gulshan-e-Iqbal (2), Gadap (3), and Hyderabad (1) district in Sindh province.

B SL prevalence model

Sabin prevalence in acute flaccid paralysis surveillance cases (AFP) is strongly dependent upon the timing of supplemental immunization activities (SIAs; Kroiss et al. 2017). Sabin prevalence rises rapidly in the first few days after SIAs and then gradually returns to baseline levels within 50 days after a campaign. Prevalence between SIAs (baseline prevalence) is likely driven by persistent routine immunization (RI) for children under 1 year of age and secondary transmission. The pattern of Sabin infections post-SIA has implications for the pattern of ES detection of Sabin.

We estimated the prevalence of Sabin 1, 2, and 3 on the collection date of each ES sample using a modified version of the models described in Kroiss et al. 2017. Specifically, we used a piecewise modeling approach that captured the rapid rise to peak prevalence after SIAs with a linear function followed by an exponential decay. Our model accounted for district level differences in the duration of Sabin detection after SIAs and baseline detection rates (likely from differences in RI rates). We accounted for overdispersion in the outcomes using a Beta-Binomial likelihood to estimate the components of the detection probability $p_{ijt}$ indexed by country $i$, Sabin type $j$, and $t$ days from the last SIA, that is

$y_{ijt}\sim BetaBinomial\left( n_{ijt}, p_{ij}\left( t \right), \theta_{ij} \right)$

$p_{ij}\left( t \right)=\alpha_{ij}+\beta_{ij}*\min\left( 1,\frac{t}{\delta_{ij}} \right)*e^{-\gamma_{ij}*max(0,t-\delta_{ij})}$

Alternatively, $p_{ij}\left( t \right)$ may be written

$$p_{ij}\left( t \right)= \left\{ \begin{matrix} \alpha_{ij}+\beta_{ij}\cdot\frac{t}{\delta_{ij}} for 0\leq t<\delta_{ij}; \\ \alpha_{ij}+\beta_{ij}e^{\left\{ -\gamma_{ij}\left( t-\delta_{ij} \right) \right\}} \mathrm{for}t\geq\delta_{ij}. \end{matrix} \right.$$

Here $y_{ijt}$ represents the number of SL positives of $n_{ijt}$ NP-AFP cases, and $\theta_{ij}$ the overdispersion. $\alpha_{ij}$ captures baseline detection rates (likely coming from routine immunization), $\beta_{ij}$ describes the shape of the curve, and $\delta_{ij}$ describes the time from SIA to peak Sabin prevalence. We estimated model parameters with a Bayesian approach, with posterior samples generated through Markov Chain Monte Carlo (MCMC) as implemented in STAN v2.10.0 via the rstan package [1] in R v3.4.1 [2].

$\alpha_{ij}$, $\gamma_{ij}$, and $\theta_{ij}$ parameters were constrained to be between 0 and 1. These terms were hierarchically specified as

$$\mathrm{logit}\alpha_{ij}\sim N\left( a,\sigma_{\alpha} \right)$$

$$\mathrm{logit}\gamma_{ij}\sim N\left( g,\sigma_{\gamma} \right)$$

$$\mathrm{logit}\theta_{ij}\sim N\left( d,\sigma_{\theta} \right)$$

$$a\sim N\left( 0,5 \right)$$

$$g\sim N\left( 0,5 \right)$$

$$d\sim N\left( 0,5 \right)$$

$$\pi\left( \sigma_{\alpha} \right)\propto1$$

$$\pi\left( \sigma_{\gamma} \right)\propto1$$

$$\pi\left( \sigma_{\theta} \right)\propto1$$

The priors on the conditional (logit) medians $a$, $g$, and $d$, are fairly permissive despite not appearing particularly diffuse with standard deviations of 5 each. Improper, flat priors were used for the standard deviations for the random terms.

We set a uniform prior on $\delta$with boundaries at 2.5 and 7.5, i.e. $\gamma\sim Uniform(2.5, 7.5)$.

$\beta_{ij}$ was carefully parameterized to ensure that $p_{ij}\in(0,1)$. Noting that $\beta_{ij}\in\left( 0,1-\alpha_{ij} \right)$, we specify as

$$\beta_{ij}=\left( 1-\alpha_{ij} \right)\cdot\phi_{ij}$$

$$\phi_{ij}\sim N\left( f,\sigma_{\phi} \right)$$

$$f\sim N\left( 0,5 \right)$$

$$\sigma_{\phi}\propto1$$

Using these priors, we ran four sampler chains with a burn-in of 500 and 1000 monitored iterations. We assessed model convergence by examining the effective number of samples and the point scale reduction statistics [3,4].

Note that this modeling produces estimates of Sabin prevalence for the geography specified based on time since SIA. It does not model how the infection trajectory might change over time for different SIA. Thus the eventual regression of ES outputs is on a modeled “typical” infection trajectory for a given geography relative to the time since last SIA.

A posterior point estimate as a function of time since SIA was produced by the posterior mean at each time point, i.e.

$$\tilde{p}_{ij}\left( t \right)=\frac{1}{M}\sum_{m=1}^{M} p_{ij}^{\left( m \right)}\left( t \right)$$

where $p_{ij}^{\left( m \right)}\left( t \right)$ is the $m$-th posterior sample constructed from the constituent parameters from that sample, of $M$ posterior samples.

C Delayed peak detection rate in ES relative to AFP

We explored whether Sabin detection rates in ES may be delayed relative to AFP. This could occur since ES may detect secondary transmission in populations typically not sampled by AFP (children >5 years old). We examined this possibility by comparing the log-likelihoods of the ES site sensitivity model fit to ES data for which the Sabin prevalence estimates were based on times since SIA that that had been artificially time shifted by 0 to 25 days. That is, rather than use the prevalence estimated from NPAFP data for the same day, we mapped the ES observation day to a prior time point parameterizing the Sabin prevalence curve. Specifically, we shifted times since SIA for each ES sample such that

$$s=t-\eta\cdot min(1,\frac{t}{\delta+\eta}).$$

Here, $s$ represents the time input used to extract the prevalence from the Sabin prevalence model ($p_{ij}\left( s \right)$) $t$ represents the observation time of ES, $\delta$ represents the original time to peak prevalence in the Sabin prevalence model, and $\eta$ represents the number of days to shift the peak of the data. The structure is such that the shift is linear in time from zero to up to a maximum shift of $\eta$. We used the shifted times since SIAs to estimate the Sabin prevalence for each ES sample. An example of the time shifted data is shown in Fig A & B.


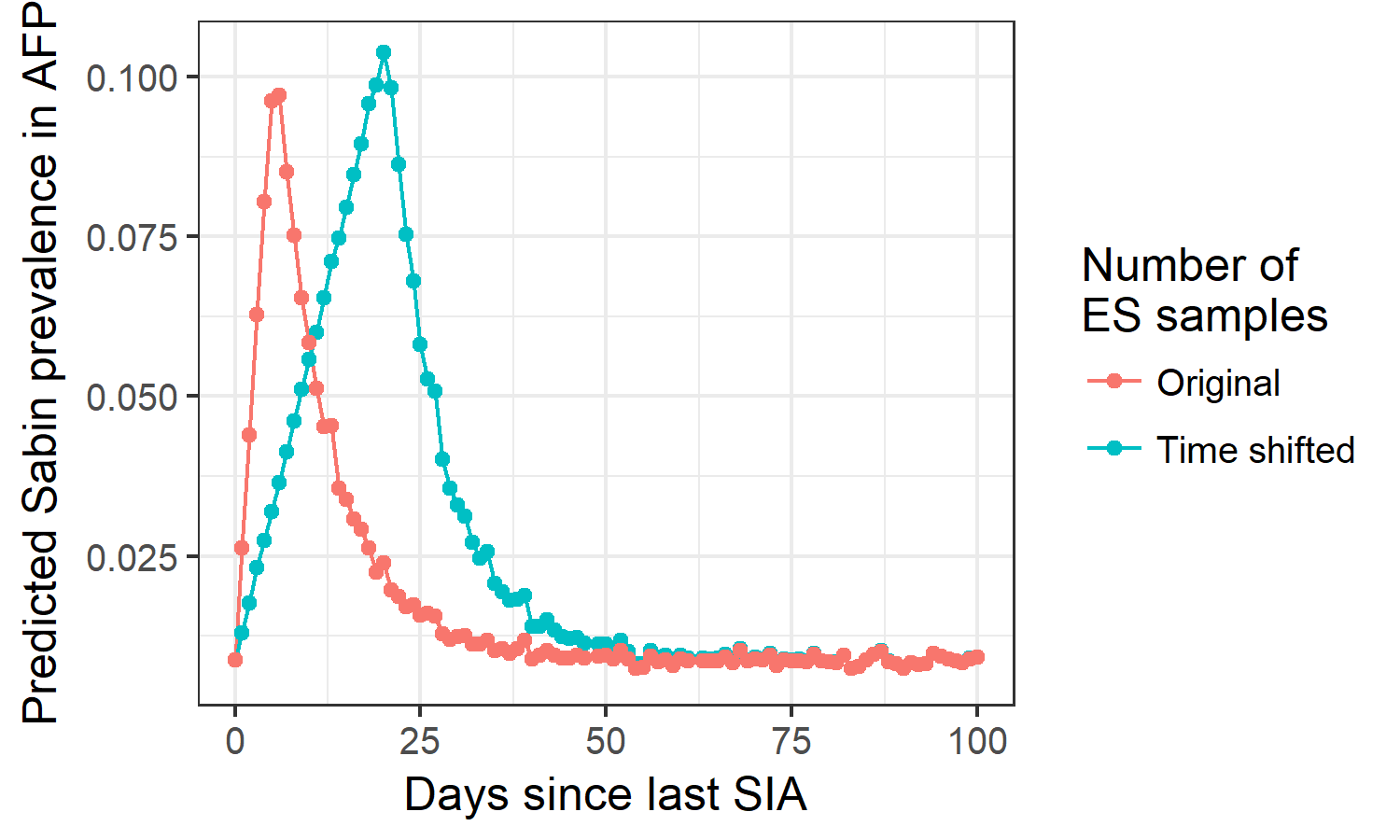


Fig A – Artificially shifting the time since SIA for ES samples allows for a delayed peak in Sabin prevalence predictions that better fit with the delayed peak in ES detection rates relative to AFP. The data show are for SL2 in Pakistan, and each point reflects a country level mean. The time shifted line reflects a time shift of 15 days.


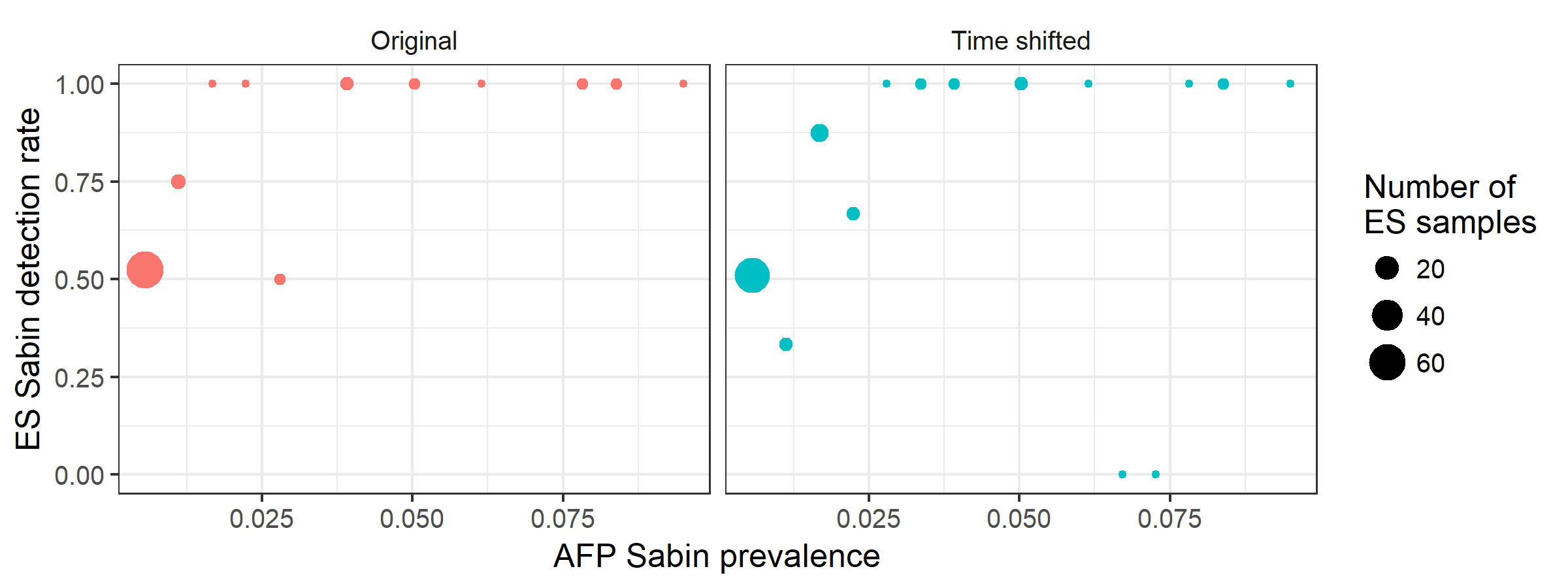


Fig B – Artificially shifting the time since SIA for ES samples and thus the prevalence predictions slightly alters the data used in the ES sensitivity model. While the ES detections themselves (0’s and 1’s) remain the same, the prevalence is shifted for each point. To display these changes, we aggregated detection rates in bins of 0.006 prevalence. The data shown are for SL2 in Pakistan for the MR ES site in Lahore district.

We analyzed the time shifted data using a simplified version of the ES site sensitivity model described in the main text. Specifically, we modeled each virus type in each country separately using a binomial regression with complementary log-log (cloglog) link to estimate the components of the detection probability $p_{jk}$ indexed by ES site *j*, and sample k, that is

$y_{jk}\sim Bern\left( p_{jk} \right)$

${\mathrm{cloglog}(p}_{jk})=\eta+\beta*\log\left( x_{jk} \right)+u_{j}$

$\mathrm{cloglog}\left( p_{jk} \right)=\log\left( -\log\left( 1-p_{jk} \right) \right)$

Here, $y_{jk}$ represents positive (y=1) or negative (y=0) detections of virus, $\eta$ is the intercept, $\beta$ is a coefficient for the time-shifted virus prevalence covariate $x_{jk}$, and $u_{j}$ is a random effect for ES site level detection rates. We estimated model parameters with a generalized linear mixed-effect model using the glmer function in the lme4 package [5] in R v3.4.1 [2]. We repeated this analysis for a range of time delays (0-25 days) and compared the log-likelihoods between the models (Fig C).

Comparisons of the log-likelihoods indicated that delayed peaks in Sabin prevalence in ES relative to AFP were supported in Pakistan, but there was little support for delays in Afghanistan (Fig C). In Afghanistan, log-likelihoods were highest with no time shift for SL1, a 5 day delay for SL2, and a 5 day delay for SL3. However, the changes in log-likelihood between the original data and the peak likelihood were small (<2). In Pakistan, log-likelihoods were highest with an 8 day delay for SL1, a 15 day delay for SL2, and an 11 day delay for SL3. The differences in log-likelihood between the peak and that for the original data were all greater than 12.

We compared the ES site rankings between the full ES site sensitivity model fit to the original data and the data with delayed peaks (Fig D). The changes in rankings were minimal across all virus types and did not significantly affect the rankings.


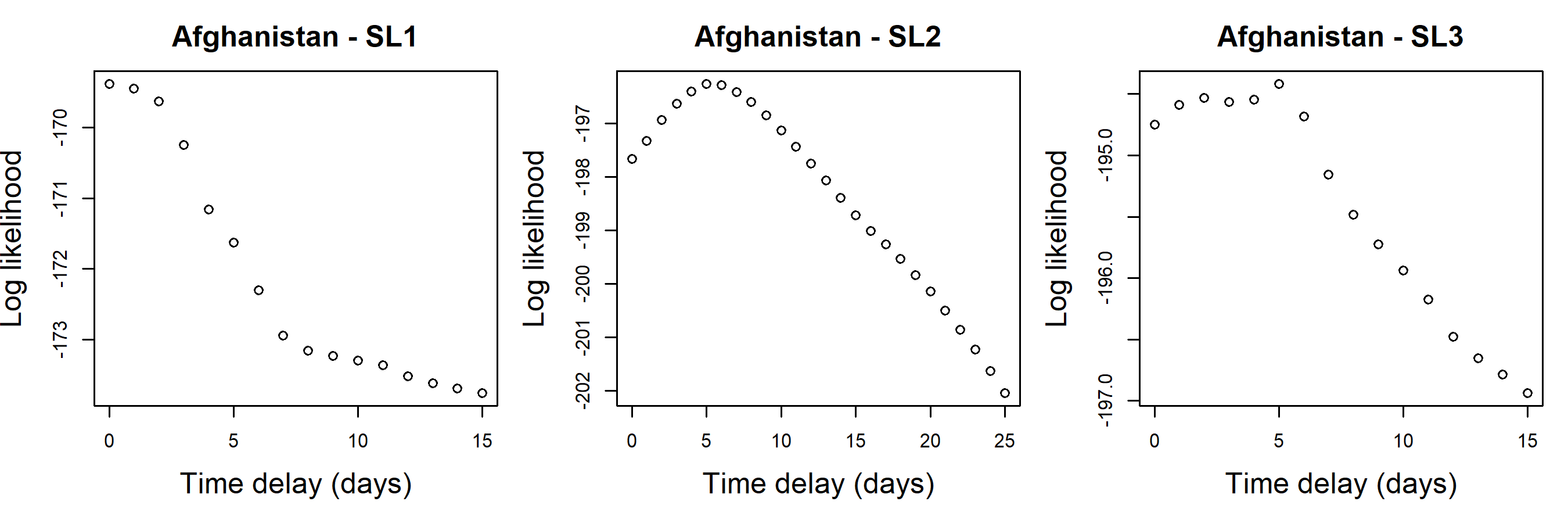


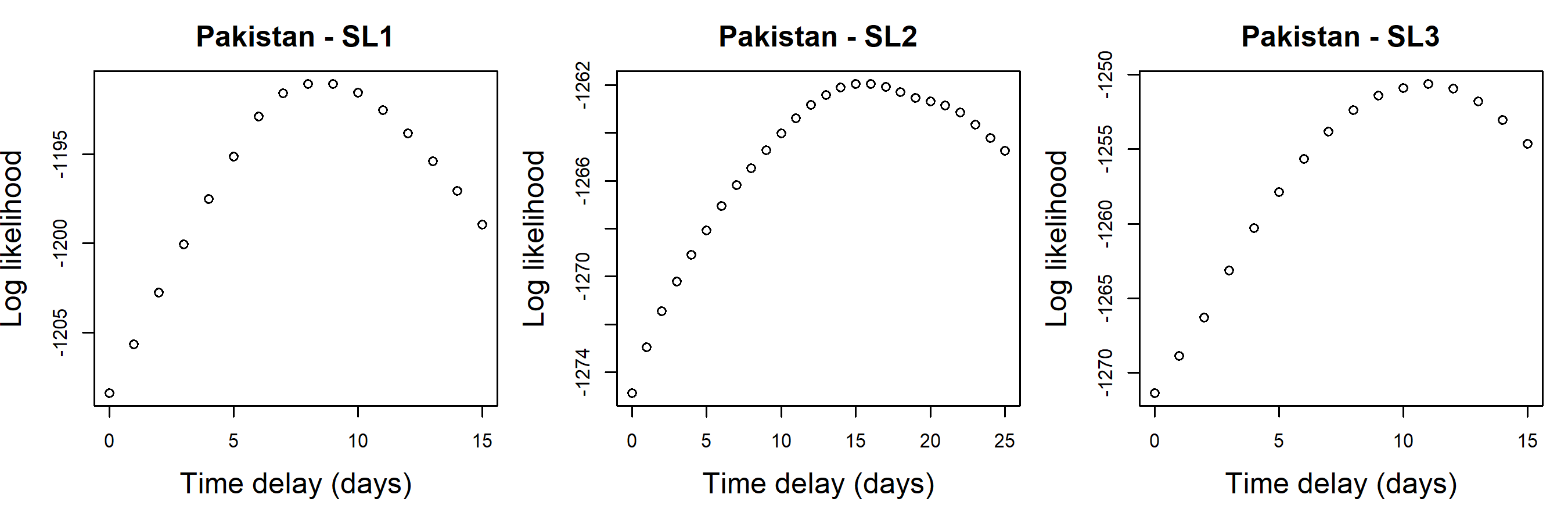


Fig C – Log-likelihoods from models fit to the ES data with artificially shifted time since SIA (and thus virus prevalence estimates). Each panel shows the results for a single Sabin-like (SL) type for each country.


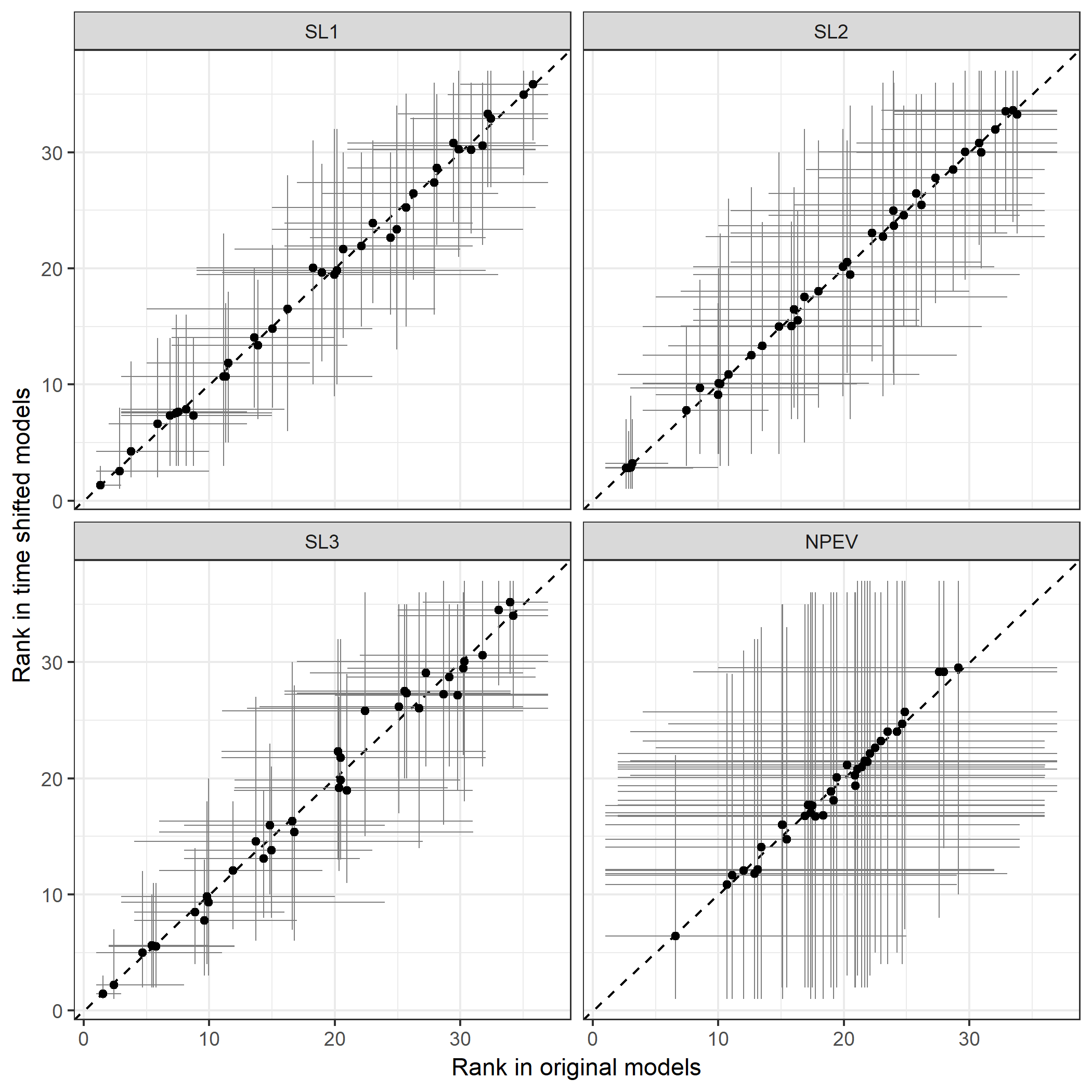


Fig D – Comparisons of model rankings of each of the ES sites in Pakistan based on the original and time shifted models. The dashed line represents a 1:1 relationship. The vertical and horizontal grey bars indicate 95% confidence intervals across the 500 model posteriors.

Note that we did not attempt many alternative parametric forms for a potential time-shift mapping, though certainly others exist, including those that may be more biologically motivated.

D NPEV prevalence model

To estimate the prevalence of non-polio enteroviruses (NPEV) on the collection date of each ES sample, we modeled the detection rate of NPEV in AFP cases after 2009 at the one month time scale (Fig E). We estimated prevalence at the district level in Pakistan and the province level in Afghanistan. We accounted for spatial heterogeneity and seasonal fluctuations in prevalence using a Binomial regression to estimate the components of the detection probability $p_{ijkl}$ indexed by province (Pakistan) or UN region (Afghanistan) $i$, district (Pakistan) or province (Afghanistan) *j*, month of the year $k$, and time period $l$. Month of the year is an index repeated each year, while time period refers to the unrepeated monthly index. We will subsequently describe the model for Pakistan; the description holds for Afghanistan with regions in place of provinces and provinces in place of districts.

$y_{ijkl}\sim Binomial\left( n_{ijkl}, p_{ijkl} \right)$

$$p_{ijkl}=\beta_{0}+b_{i}+c_{ij}+u_{k}+v_{ik}+\kappa_{l}+\omega_{il}+\epsilon_{ijl}$$

Here $y_{ijkl}$ represents the number of NPEV positives of $n_{ijkl}$ NP-AFP cases. Model terms were included to allow for structured variation. Effects are included for the overall (time-invariant) “level” of NPEV detection, including a country intercept $\beta_{0}$, a province random intercept $b_{i}$ describing variation from the country level, and a spatially structure district random intercept $c_{ij}$ using the BYM [6]. For seasonality (period of 1 year), cyclic random walks (which create temporal structure while returning to the starting point) were included at the country level and province level, $u_{k}$ and $v_{ik}$ respectively [7]. This structure may be thought of as a country-level seasonality for which provincial variation in seasonality is allowed. Finally there are three levels of autoregressive errors with lag 1 (i.e. AR(1)) at the country, province, and district levels, $\kappa_{l}$, $\omega_{il}$, and $\epsilon_{ijl}$ respectively. These terms are meant to allow for observation level errors that still may have some spatiotemporal structure, which might be expected for observations of a group of transmitted pathogens. We estimated model parameters using a Bayesian approach using the INLA package v0.0-1468872408 [8,9] in R v3.4.1 [2].


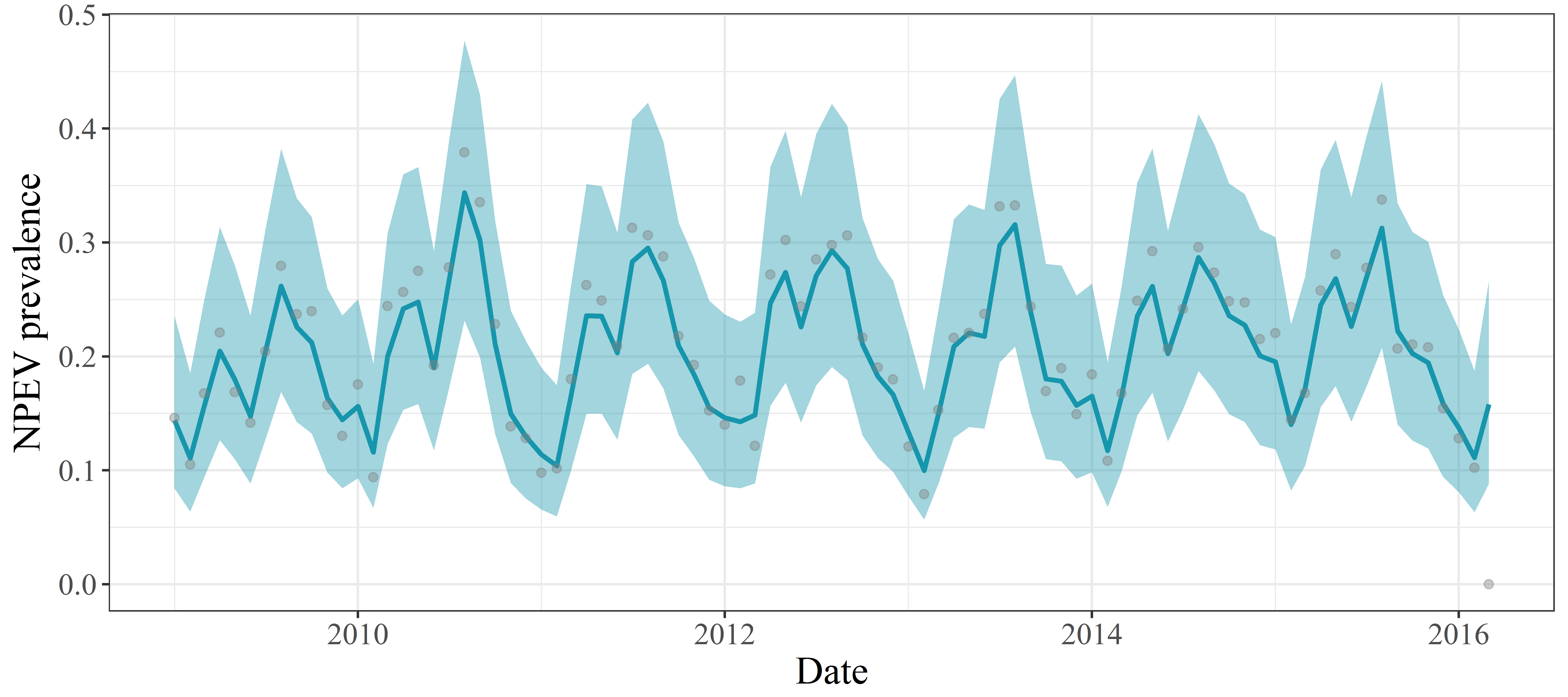


Fig E: NPEV prevalence estimates for Pakistan. NPEV prevalence displays distinct seasonal patterns that typically peak from April to October with slight declines each June. The grey dots represent observed rates of NPEV detection from pre-cessation AFP data. The solid blue line represents mean model predictions and the blue ribbon represents 95% credible intervals. Our models were run at the district level, but for simplicity we present the mean patterns across all of Pakistan.

E ES site sensitivity model sampling

Environmental samples result in presence/absence of the three serotypes of polio and non-polio enterovirus. Separate analysis could conceivably be executed for each separately, but a composite sensitivity would be impaired by lack of accounting for correlation between the serotypes on a site and sample level. However, the censoring of NPEV results when polioviruses are detected on the RD flask necessitated that we model Sabin and NPEV separately. Thus, we constructed two models: a joint model for Sabin 1, 2, and 3, and a separate model for NPEV only.

We begin by describing the joint ES site sensitivity model for Sabin 1, 2, and 3. To account for correlation on the site (sensitivity) and observation (noise and inter-type interference) level, we included random effects at each of these levels. In matrix form, indexed by site $i$, observation $j$, and serotype $s$, we have

$$Y_{ijs}|q_{ijs}\sim Bernoulli\left( q_{ijs} \right)$$

$$\log\left( -\log\left( 1-q_{ijs} \right) \right)=\beta_{0}+b_{i}+\beta_{1}\log x_{ij}^{T}+\epsilon_{ijs}$$

$$b_{i}\sim MVN\left( 0,\Sigma_{1} \right)$$

$$\epsilon_{ij}\sim MVN\left( 0,\Sigma_{2} \right)$$

The transformation of $q_{ijs}$ is the cloglog link function. In the formulation we define $b_{i}$ as the site sensitivity by serotype, noting that is the time-invariant component of sensitivity unique to the site, i.e.

$$q_{ijs}=1-exp \left\{ -\exp\left\{ b_{is} \right\}x_{ijs}^{\beta_{1s}}\exp\left\{ \beta_{0s}+\epsilon_{ijs} \right\} \right\}.$$

To estimate, we use a Bayesian approach such that $b_{i}$ are estimated explicitly. Flat priors were used for the intercepts and regression coefficients $\beta_{0}$ and $\beta_{1}$. Wishart priors are used for $\Sigma_{1}^{-1}$ and $\Sigma_{2}^{-1}$ with $\Sigma_{1}^{-1}\sim Wishart\left( 7,I_{3} \right)$ and $\Sigma_{2}^{-1}\sim Wishart\left( 7,I_{3} \right)$. These are the default priors used in the software used for estimation.

We also ran a site sensitivity model for NPEV only. This model has the same form as the one above for Sabin 1, 2, and 3, but without the site and sample level correlations between the serotypes ($b_{i}$ and $\epsilon_{ij}$).

We estimated model parameters using a Bayesian approach using the INLA package v0.0-1468872408 [1][8,9] in R v3.4.1 [2]. We drew 1000 posterior samples for each parameter to generate credible intervals for subsequent analyses similarly using INLA.

F ES site sensitivity results


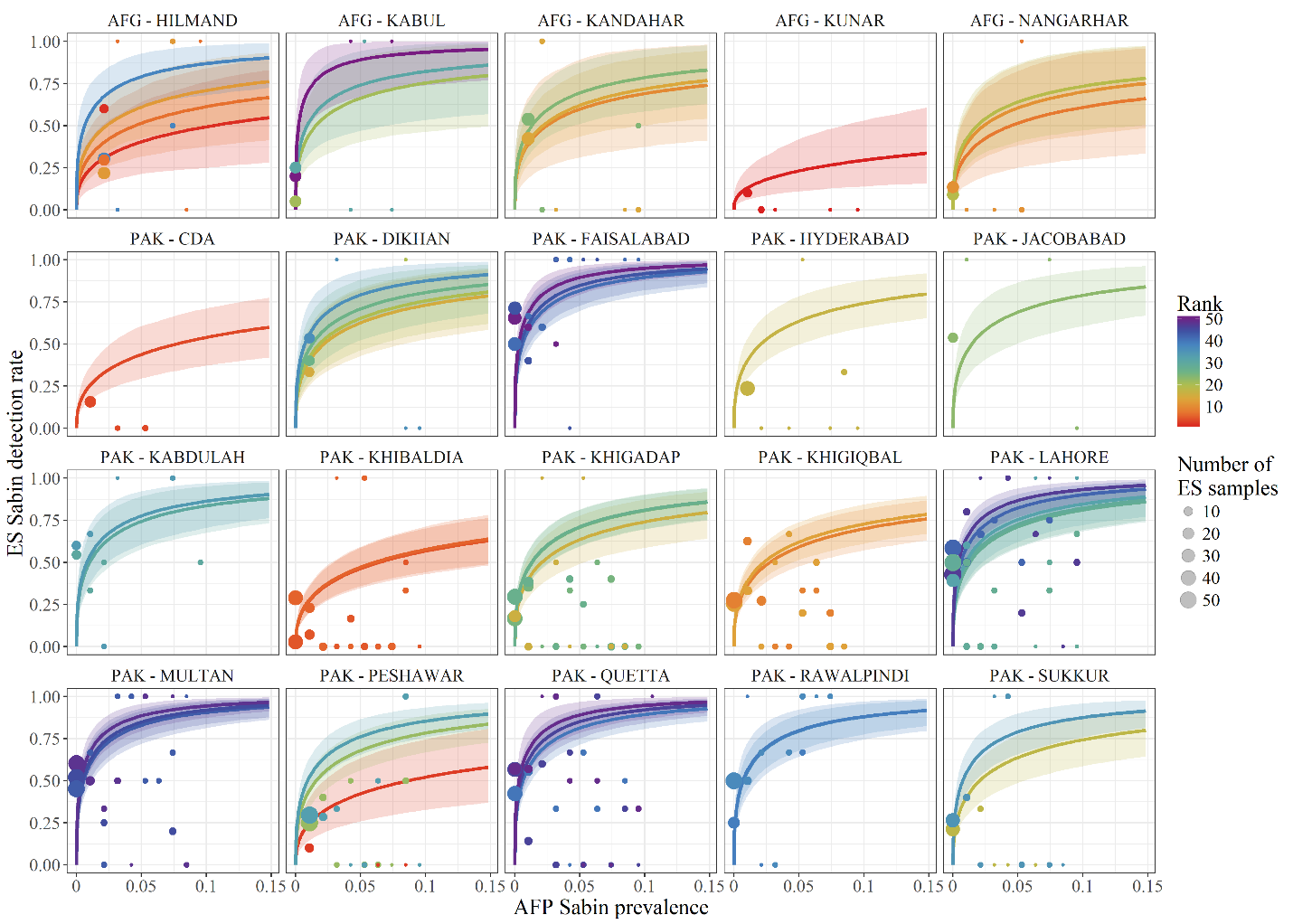
Fig F: ES sensitivity curves for Sabin 2 for each ES site in Pakistan. The dots represent observed rates of Sabin detection from pre-cessation ES data and prevalence estimates based on pre-cessation AFP modeling. The dots are scaled to the number of samples. The solid lines represents mean model predictions for each ES site and the ribbons represent 95% credible intervals. Data and model estimates are colored by ES site from least sensitive (red) to most sensitive (purple).


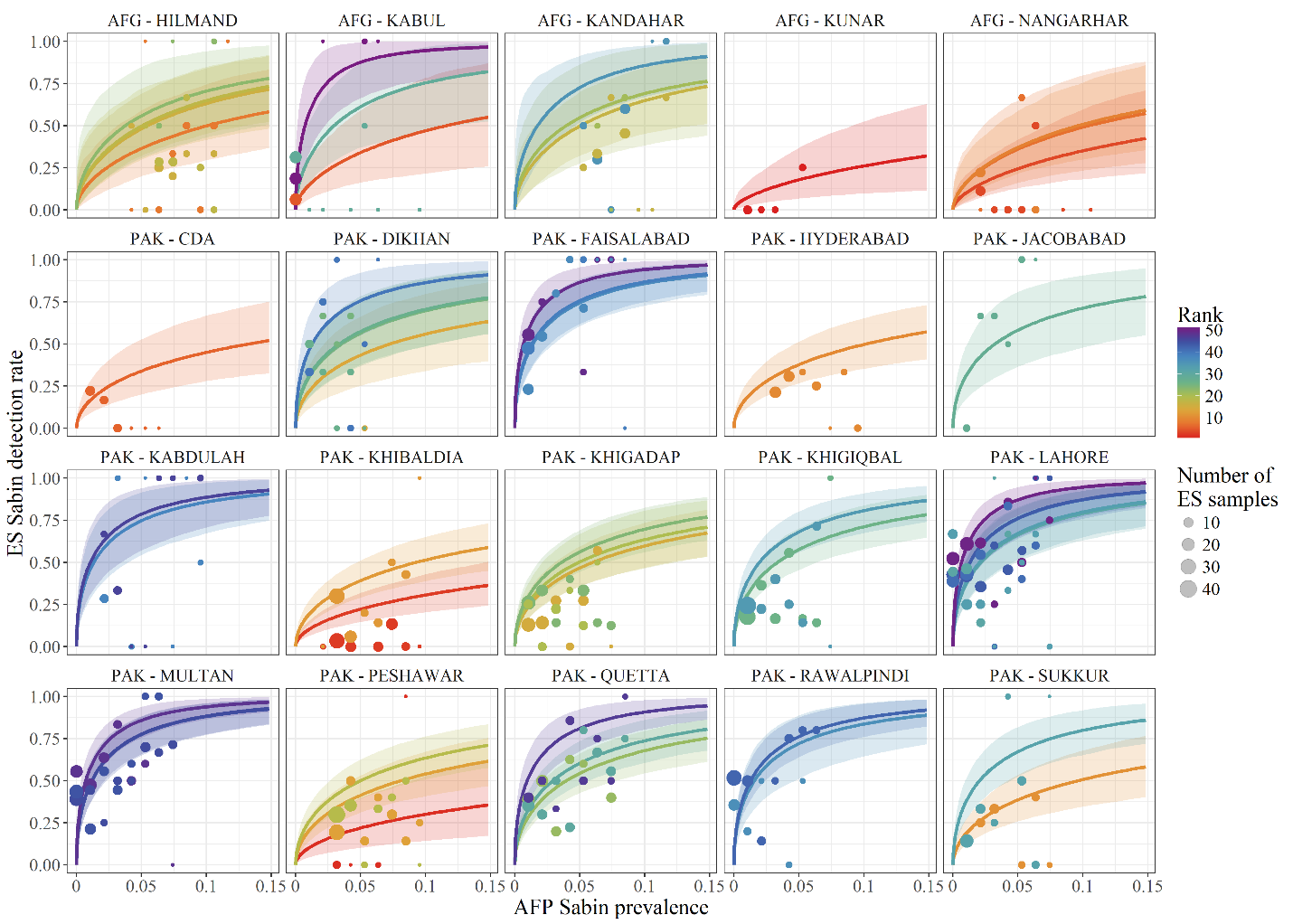


Fig G: ES sensitivity curves for Sabin 3 for each ES site in Pakistan. The dots represent observed rates of Sabin detection from pre-cessation ES data and prevalence estimates based on pre-cessation AFP modeling. The dots are scaled to the number of samples. The solid lines represents mean model predictions for each ES site and the ribbons represent 95% credible intervals. Data and model estimates are colored by ES site from least sensitive (red) to most sensitive (purple).


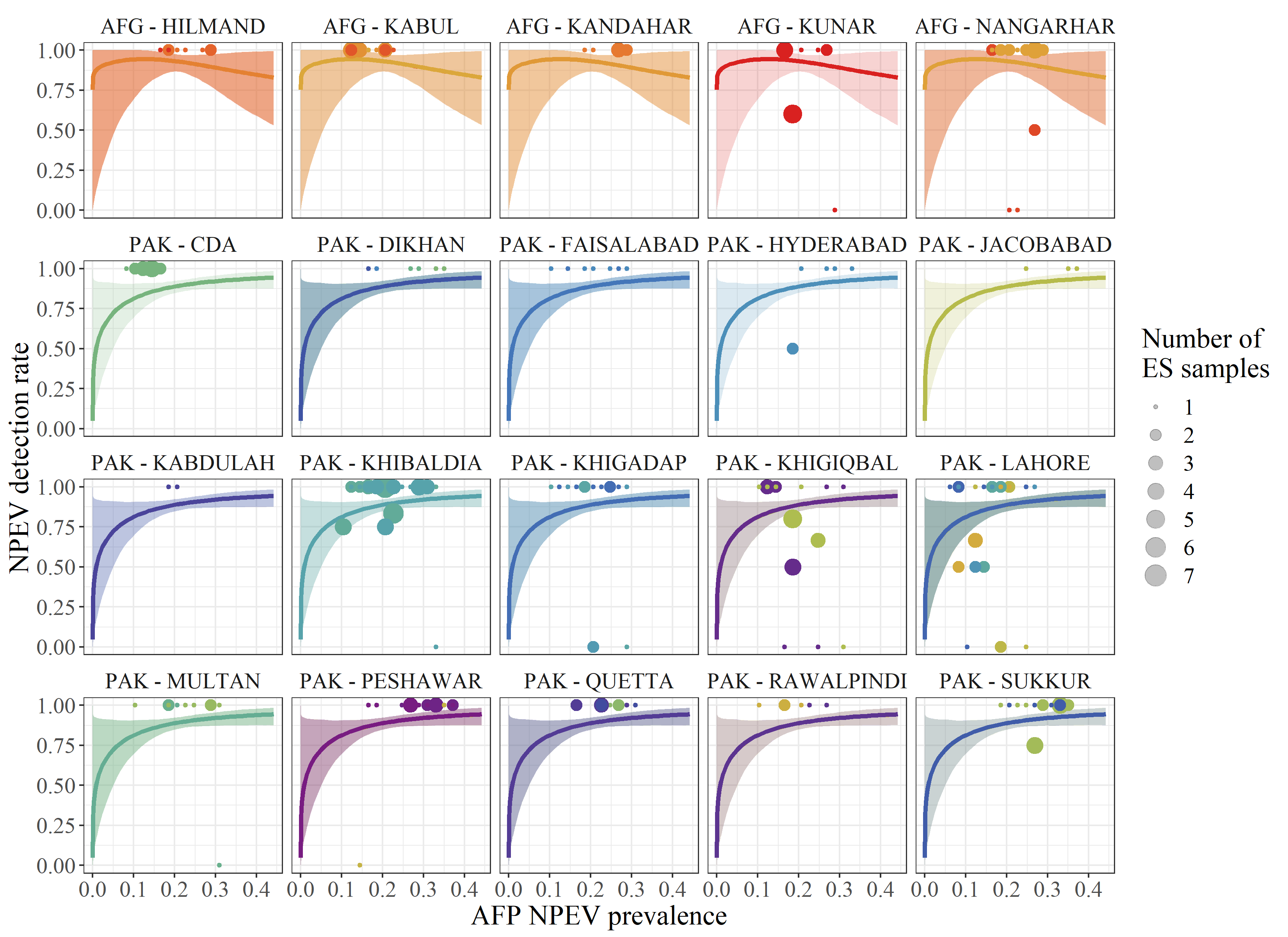


Fig H: ES sensitivity curves for NPEV for each ES site in Pakistan. The dots represent observed rates of NPEV detection from pre-cessation ES data and prevalence estimates based on pre-cessation AFP modeling. The dots are scaled to the number of samples. The solid lines represents mean model predictions for each ES site and the ribbons represent 95% credible intervals. Data and model estimates are colored by ES site from least sensitive (red) to most sensitive (purple).

The NPEV sensitivity models were too underpowered from limited sample sizes to distinguish sensitivity differences between ES sites (Fig H). Specifically, none of the site sensitivity parameters, $b_{i}$, were significantly different from each other. The NPEV regression coefficients, $\beta_{1}$, included negative estimates for both Afghanistan and Pakistan, but only Afghanistan had a mean negative estimate. Negative estimates are not logically supported, but neither country had regression coefficients that were significantly different than zero. More data, or access to long-term flask level data could provide better regression estimates for NPEV.

G Flask-level interference analysis

Wild type 1 poliovirus (WPV1) is competitively superior to SL viruses in the cell culture step of analysis, thereby potentially interfering with the detection of Sabin-like viruses (SL1/2/3). If so, ES site sensitivity based on SL and NPEV viruses could be biased due to this interference. However, actual detection depends on concentration and assay sensitivity, so it actual interference may vary in practice. We examined the evidence for interference using flask-level ES data 2017 data from Pakistan. There are two directions of this analysis, first analyzing the sample-level results and second the flask level results.

## Sample-level results

At the sample level, NPEV was the most isolated virus type followed by SL3, SL1, WPV1, SL2, and cVDPV2 (Table A). We may roughly explain this in that NPEV prevalence is quite high all year round in Pakistan compared to SL viruses, which tend to spike after campaigns, rapidly dwindling to levels associated with routine immunization. SL3 and SL1 are also very likely to occur due to many bOPV campaigns, the opposite true of any type 2 due to OPV2 cessation and the limited geographic scope of VDPV2 detection and mOPV2 response. In contrast with type 2, WPV1 has been identified in multiple geographies in Pakistan in 2017 and is subsequently more frequently detected.

Table A – Virus isolation rates in Pakistan ES samples from 2017, N = 417 samples

| **Virus Type** | **Positive** | **Proportion** |
| --- | --- | --- |
| **WPV1** | 61 | 0.15 |
| **SL1** | 208 | 0.50 |
| **SL2** | 44 | 0.11 |
| **SL3** | 261 | 0.63 |
| **VDPV2** | 5 | 0.01 |
| **NPEV** | 300 | 0.72 |

We examined the sample level correlations in detection rates in ES among virus types. Use of correlation is not the most elegant treatment of binary data. However, when two virus types are positively correlated on the sample level, it implies that co-occurence may be more likely than interference. Further it implies that a simple treatment of the whole set of data as independent samples will not find evidence of interference. This is true because positive correlation implies that

$Cov\left( X_{ij}, X_{ik} \right)=p_{jk}-p_{j}p_{k}>0\to p_{jk}>p_{j}p_{k}$

where $X_{ij}$ is the presence or absence of virus type *j* in environmental sample *i*, $p_{jk}$ the probability that the two types co-occur, and $p_{j}{,p}_{k}$ are probability of types *j, k*.

Table B – Correlation in detection rates in ES among virus types.

|  | **WPV1** | **SL1** | **SL2** | **SL3** | **VDPV2** |
| --- | --- | --- | --- | --- | --- |
| **SL1** | 0.03 |  |  |  |  |
| **SL2** | 0.28 | 0.13 |  |  |  |
| **SL3** | 0.15 | 0.35 | 0.15 |  |  |
| **VDPV2** | 0.02 | 0.07 | -0.04 | 0.09 |  |
| **NPEV** | -0.27 | -0.31 | -0.32 | -0.41 | -0.08 |

As we can see, the only negative correlations are with NPEV with all other viruses (Table B). This almost has to be true due to the nature of NPEV reporting: RD positive cells must be positive with NPEV only or they are not reported as positive for NPEV*.* In fact, in the data set there is no example of an RD flask positive for both NPEV and any poliovirus.

The implication is that using overall averages, WPV does not apparently interfere with reporting of Sabin-like (SL) virus. The largest positive correlation is with SL1 and SL3, which is understandable in that they co-occur in the bOPV and thus have common origin. We may modify this result by conditioning on the sites that report at least 1 WPV for a more relevant comparison

We forgo a formal model treatment, instead opting for a “pooled” correlation. Briefly, this is done by computing a pooled covariance estimate -- essentially a weighted average of site-level covariance matrices -- for sites with any WPV1 detection in 2017 and then converting to correlation matrix. That is, the pooled estimate for the correlation of types $j$ and $k$, $\tilde{\rho}_{jk}$, computed over the $n_{i}$ samples from site $i$ is

$$\tilde{\rho}_{jk}=\frac{\sum_{i=1} \sum_{s=1}^{n_{i}} \left( x_{ijs}-\bar{x}_{ij} \right)\left( x_{iks}-\bar{x}_{ik} \right)}{\sqrt{\sum_{i=1} \sum_{s=1}^{n_{i}} \left( x_{ijs}-\bar{x}_{ij} \right)^{2}}\sqrt{\sum_{i=1} \sum_{s=1}^{n_{i}} \left( x_{iks}-\bar{x}_{ik} \right)^{2}}}.$$

Table C – Pooled correlation of types for sites with any WPV1 in 2017.

|  | **WPV1** | **SL1** | **SL2** | **SL3** | **VDPV2** |
| --- | --- | --- | --- | --- | --- |
| **SL1** | -0.06 |  |  |  |  |
| **SL2** | 0.19 | 0 |  |  |  |
| **SL3** | 0.18 | 0.29 | 0.16 |  |  |
| **VDPV2** | -0.06 | -0.01 | -0.29 | 0.07 |  |
| **NPEV** | -0.24 | -0.17 | -0.27 | -0.34 | -0.01 |

The absence of high magnitude negative correlations between WPV1 and SL viruses is noteworthy, with the only negative estimate between WPV1 and SL1 being particularly small in magnitude. One observation is that catchments with highly successful OPV vaccination (bOPV) should be at less risk for WPV1 circulation and subsequent detection of WPV1. In fact, WPV1 in absence of OPV1 might say something about the level of vaccination in that area.

In summary of the sample level results: 1) across all samples, at the sample level there is little evidence of WPV1 interference with SL and VDPV virus; correlations are actually non-negative; 2) restricting to sites with WPV1 detection, there remains scant evidence of WPV1 interference. These results should not be interpreted to mean that WPV cannot interfere with SL virus – they must at some level of differential concentration -- but that in the observed field conditions we cannot find strong evidence of it.

## Flask level analysis

We examined the evidence for whether polio types interfere with each other on a flask level. Initial examination of the flask level data indicated that it is not uncommon for a L20B flask to be positive for multiple types of poliovirus, including WPV1. Based on this observation, we devised a permutation test to compare the observed rate of WPV1 and SL1 detection in the same flask compared to what would be possible.

For our permutation procedure, we began by selecting two viruses to be compared. We focused on the following contrasts: WPV1 vs SL1, WPV1 vs. SL3, and SL1 vs. SL3. We then calculated the number of flask-level co-occurrences across all samples. We subset the ES samples to those with at least one positive flask for each of the types, not necessarily the same flask. For each sample and virus type, we randomly shuffled the flask levels. For example, while {1,0,0,0,0} may have been observed for a virus, {0,1,0,0,0} may result from this shuffle; this could have been an equally likely outcome under the null hypothesis. We computed the number of co-occurrences from this shuffled data and repeated the procedure 2000 times. We contrasted the observed number of co-occurrences to the results from the permutation approach; an extreme result suggests the null hypothesis is incorrect. We used a one-sided test, as the alternative hypothesis is one of interference.

Note that on a sample level the conditional distribution is Hypergeometric, e.g. with $m_{j}$positive flasks of type $j$ and $m_{k}$ of type $k$, and $Y_{jk}$ the number of co-ocurrences we have under independence

$$P\left( Y_{ijk}=m_{jk}|m_{j},m_{k} \right)=\frac{\left( \begin{matrix} m_{j} \\ m_{jk} \end{matrix} \right)\left( \begin{matrix} {5-m}_{j} \\ m_{k}-m_{jk} \end{matrix} \right)}{\left( \begin{matrix} 5 \\ m_{k} \end{matrix} \right)}=\frac{\left( \begin{matrix} m_{k} \\ m_{jk} \end{matrix} \right)\left( \begin{matrix} {5-m}_{k} \\ m_{j}-m_{jk} \end{matrix} \right)}{\left( \begin{matrix} 5 \\ m_{j} \end{matrix} \right)}$$

This may be motivated by placing $m_{j}$ positives among the 5 flasks and then randomly drawing $m_{k}$ from the collection of these positive and negative flasks.

This approach relies on two key assumptions. First, poliovirus types do not (practically) interfere with each other on a flask level. That is, we may assume that given typical inoculations of virus A and virus B into the same flask, results for the two types for that flask are independent. Second, given the presence of a poliovirus type, presentation in the 5 flasks are independent and identically distributed. This implies that if, for example, 3 of 5 flasks are positive for virus A, any selection of 3 of 5 flasks would have been equally likely. In other words, given an ES sample, each flask is equally likely to be positive for WPV1 and each flask is equally likely to be positive for SL1, and further a flask positive for WPV1 has no effect on whether SL1 will be isolated from that flask.

We found little evidence against the null hypothesis outlined above, that is conditional independence of flask level presentation of virus and non-interference/independence of virus type (Fig I). Specifically, none of the virus pairs indicated significanst differences between the observed amount of overlap and that of the permutation test for either WPV1 vs. SL1 (p-value = 0.43), WPV1 vs. SL3 (p-value = 0.79), or SL1 vs. SL3 (p-value = 0.95). This suggests that under typical conditions of concentration in Pakistan in 2017 we do not see extreme interference between virus types, notably WPV1 vs. SL viruses.

Note that the assumptions of the null hypothesis are fairly strong. For example rather than each flask being equally likely to produce a positive result for a virus type, it could be that due to incomplete mixing or other lab artifacts that some flasks or more likely to produce positive results than others. In this case, the rate of co-occurrence might be expected to be higher than the assumptions that we outline.


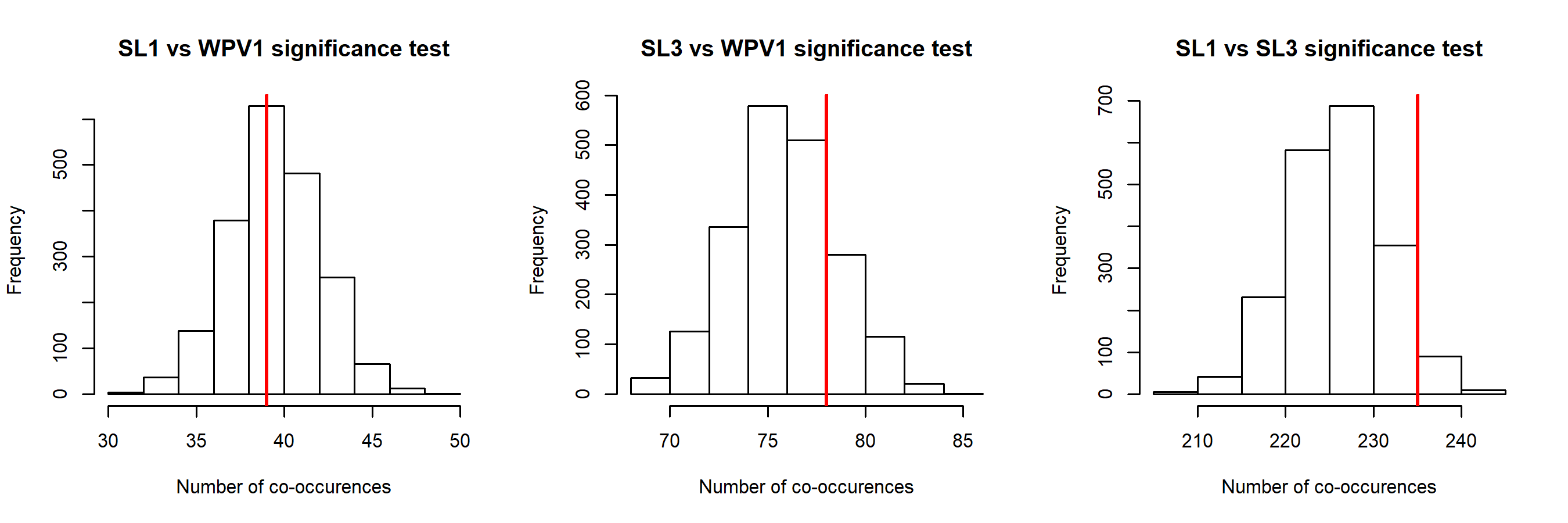


Fig I – Histograms showing the possible number of co-occurrences from the permutation procedure. The vertical red lines indicate the observed number of virus co-occurrences.

H Site information and virus detection rates

**Table D – ES site locations, detection rates, and rankings.** WPV1 detection rates and model rankings are bolded red if they were less than the median value for that respective parameter in each country. ES sites were flagged for site evaluation if both the WPV1 detection rates and model rankings were less than the median value for the respective parameter.

| **Ctry** | **Prov** | **District** | **Site** | **N** | **WPV1** | **SL1** | **SL2** | **SL3** | **Model rank** | **Flagged** |
| --- | --- | --- | --- | --- | --- | --- | --- | --- | --- | --- |
| AFG | ER | ASADABAD | MDKL | 17 | **0.06** | 0.06 | 0 | 0.12 | **1.3** | **Yes** |
| AFG | ER | JALALABAD | RDR | 26 | **0.08** | 0.12 | 0.38 | 0.19 | **3.2** | **Yes** |
| AFG | ER | BEHSUD | HDF | 9 | 0.11 | 0.22 | 0.22 | 0.33 | **4.7** | No |
| AFG | ER | JALALABAD | QSG | 26 | 0.15 | 0.08 | 0.38 | 0.38 | **5.3** | No |
| AFG | CR | CHAHARASYAB | KBG | 23 | **0** | 0.09 | 0.39 | 0.17 | **5.7** | **Yes** |
| AFG | SR | NAHRI SARRAJ | ZB | 20 | 0.2 | 0.35 | 0.45 | 0.45 | **6.9** | No |
| AFG | SR | KANDAHAR | RBT | 32 | 0.19 | 0.41 | 0.47 | 0.59 | **7.5** | No |
| AFG | SR | LASHKARGAH | BLB | 27 | 0.19 | 0.26 | 0.52 | 0.59 | 7.7 | No |
| AFG | SR | KANDAHAR | LWLA | 7 | **0** | 0.43 | 0.43 | 0.71 | 8.1 | No |
| AFG | SR | NAHRI SARRAJ | BSR | 12 | **0.08** | 0.67 | 0.17 | 0.75 | 8.4 | No |
| AFG | SR | LASHKARGAH | RDM | 27 | 0.22 | 0.3 | 0.74 | 0.59 | 10.1 | No |
| AFG | SR | KANDAHAR | KDK | 32 | 0.19 | 0.47 | 0.53 | 0.78 | 11.1 | No |
| AFG | CR | CHAHARASYAB | QZK | 23 | **0** | 0.26 | 0.43 | 0.43 | 11.4 | No |
| AFG | CR | CHAHARASYAB | KTN | 23 | **0.04** | 0.3 | 0.61 | 0.7 | 13.8 | No |
| PAK | SD | KHIBALDIA | BD-1 | 81 | **0.19** | 0.05 | 0.36 | 0.21 | **1.8** | **Yes** |
| PAK | KP | PESHAWAR | MUSAZAI | 15 | **0** | 0.07 | 0.13 | 0.13 | **2.8** | **Yes** |
| PAK | IB | CDA | SM | 25 | **0.08** | 0.12 | 0.2 | 0.32 | **3.7** | **Yes** |
| PAK | SD | KHIBALDIA | BD-3 | 73 | 0.23 | 0.26 | 0.3 | 0.36 | **5.1** | No |
| PAK | SD | SUKKUR | NS | 48 | 0.21 | 0.17 | 0.44 | 0.21 | **6.9** | No |
| PAK | SD | HYDERABAD | HC | 46 | 0.5 | 0.22 | 0.5 | 0.35 | **8.6** | No |
| PAK | SD | KHIKAMARI | GP-1 | 38 | 0.37 | 0.18 | 0.47 | 0.45 | **8.8** | No |
| PAK | SD | KHIKAMARI | GP-3 | 80 | 0.5 | 0.2 | 0.56 | 0.36 | **9.8** | No |
| PAK | KP | PESHAWAR | LM | 73 | 0.56 | 0.25 | 0.56 | 0.45 | **10.1** | No |
| PAK | SD | KHIGIQBAL | GI-1 | 82 | 0.23 | 0.27 | 0.44 | 0.45 | **11.3** | No |
| PAK | SD | KHIGIQBAL | GI-2 | 82 | 0.4 | 0.29 | 0.39 | 0.55 | **12.9** | No |
| PAK | KP | DIKHAN | BS | 18 | **0** | 0.33 | 0.44 | 0.44 | **14.2** | **Yes** |
| PAK | SD | KHIGULBERG | GP-2 | 81 | 0.58 | 0.26 | 0.57 | 0.48 | **14.3** | No |
| PAK | KP | PESHAWAR | ST | 74 | 0.77 | 0.26 | 0.62 | 0.51 | **15.7** | No |
| PAK | KP | DIKHAN | SP | 19 | **0.05** | 0.53 | 0.42 | 0.47 | **17.3** | **Yes** |
| PAK | SD | JACOBABAD | SP | 22 | 0.41 | 0.59 | 0.36 | 0.32 | **17.6** | No |
| PAK | KP | DIKHAN | MD | 19 | **0.16** | 0.37 | 0.53 | 0.53 | **17.8** | **Yes** |
| PAK | SD | SUKKUR | SC | 49 | 0.2 | 0.24 | 0.61 | 0.51 | **18.7** | No |
| PAK | PB | LAHORE | OF-3 | 41 | **0.15** | 0.37 | 0.51 | 0.56 | **18.7** | **Yes** |
| PAK | BN | QUETTA | JS | 70 | 0.31 | 0.43 | 0.66 | 0.47 | 20.5 | No |
| PAK | PB | RAWALPINDI | DD | 27 | 0.33 | 0.3 | 0.63 | 0.56 | 20.7 | No |
| PAK | PB | LAHORE | OF-2 | 40 | **0.18** | 0.4 | 0.5 | 0.55 | 20.7 | No |
| PAK | PB | LAHORE | OF-1 | 72 | **0.1** | 0.46 | 0.47 | 0.63 | 21.9 | No |
| PAK | BN | KABDULAH | AK | 19 | 0.26 | 0.53 | 0.53 | 0.84 | 24 | No |
| PAK | BN | QUETTA | JT | 70 | 0.46 | 0.46 | 0.73 | 0.56 | 25.3 | No |
| PAK | KP | DIKHAN | ZA | 19 | **0.05** | 0.47 | 0.63 | 0.68 | 25.4 | No |
| PAK | BN | KABDULAH | HP | 19 | 0.21 | 0.58 | 0.58 | 0.84 | 26.3 | No |
| PAK | PB | FAISALABAD | IR | 44 | **0** | 0.57 | 0.59 | 0.64 | 26.6 | No |
| PAK | PB | RAWALPINDI | SA | 72 | 0.47 | 0.54 | 0.58 | 0.57 | 26.7 | No |
| PAK | PB | MULTAN | SM | 70 | **0.16** | 0.49 | 0.6 | 0.6 | 28.7 | No |
| PAK | PB | LAHORE | GR | 82 | **0.06** | 0.46 | 0.67 | 0.62 | 28.9 | No |
| PAK | PB | MULTAN | KF | 70 | **0.13** | 0.5 | 0.64 | 0.64 | 30.6 | No |
| PAK | PB | LAHORE | MR | 82 | **0.06** | 0.6 | 0.61 | 0.73 | 31.1 | No |
| PAK | PB | FAISALABAD | GM | 40 | **0** | 0.7 | 0.73 | 0.65 | 32.1 | No |
| PAK | PB | FAISALABAD | GM | 40 | **0** | 0.7 | 0.63 | 0.78 | 32.2 | No |
| PAK | BN | QUETTA | SP | 44 | 0.27 | 0.57 | 0.77 | 0.77 | 32.4 | No |
| PAK | PB | MULTAN | AT | 70 | **0.14** | 0.54 | 0.66 | 0.7 | 32.7 | No |

I Potential correlates of ES sensitivity rankings

We tested for a correlation between ES sensitivity rankings and surveillance and vaccination rates using a Spearman rank correlation test across the posterior distribution of ES sensitivity rankings. Poor case surveillance rates could impact our sensitivity estimates since case surveillance data are used to estimate Sabin prevalence. To explore this issue, we used non-polio acute flaccid paralysis case detection rates (NP-AFP) which are often used to evaluate surveillance quality. NP-AFP rates are reported as the number of annual NP-AFP detections in an administrative unit (district level in Pakistan and province level in Afghanistan) per year per 100,000 children under 15 years of age. We found little evidence of poor case surveillance since even the lowest NP-AFP rates we observed were above the global standard of 2 NP-AFP cases per 100,000 children, and in fact most NP-AFP rates were several factors higher (Fig J). We also found little evidence that case surveillance rates biased our sensitivity estimates. We observed a moderate negative correlation (Spearman’s rho = -0.40, 95% CI [-0.50, -0.31]) between site sensitivity and NP-AFP rates. This is the opposite of what we would’ve expected if poor surveillance resulted in low site sensitivity.

We also found little evidence that vaccination rates impacted our sensitivity estimates since site sensitivity rankings were very weakly positively correlated (Spearman’s rho = 0.13, 95% CI [0.02, 0.24]) with the mean number of OPV doses reported by NP-AFP cases (Fig J).


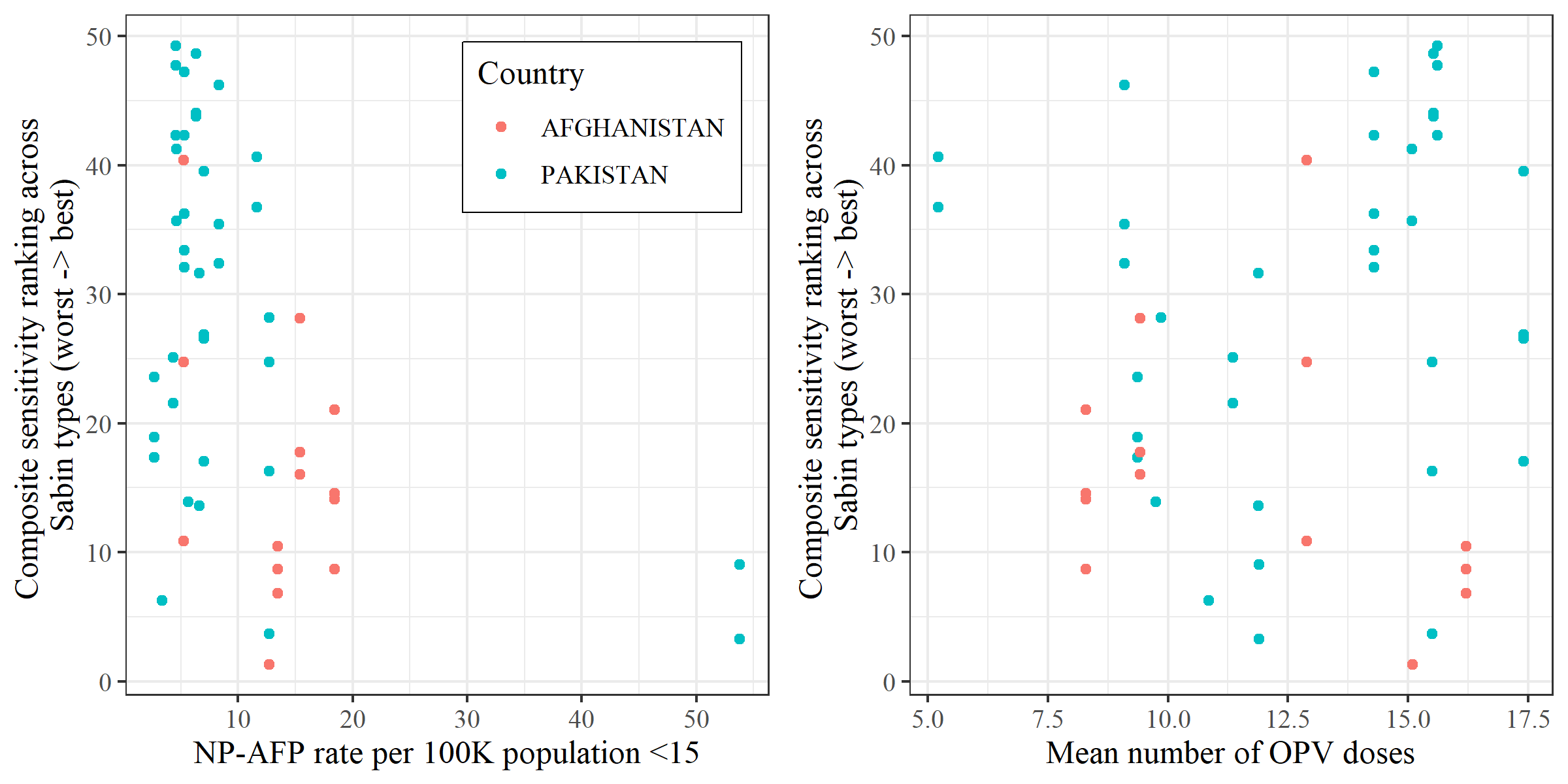


Fig J. Surveillance and vaccination rates compared to ES sensitivity estimates for each ES site. The dots represent mean sensitivity rankings (averaged across Sabin types and posteriors). Surveillance and vaccination rate data were based on NP-AFP data from 2009-2015 (prior to cessation).

References

[1] Stan Development Team. RStan: the R interface to Stan 2016.

[2] R Core Team. R: A Language and Environment for Statistical Computing 2017.

[3] Gelman A, Rubin DB. Inference from Iterative Simulation Using Multiple Sequences. Stat Sci 1992;7:457–511. doi:10.1214/ss/1177011136.

[4] Gelman A, Shalizi CR. Philosophy and the practice of Bayesian statistics. Br J Math Stat Psychol 2013;66:8–38. doi:10.1111/j.2044-8317.2011.02037.x.

[5] Bates D, Mächler M, Bolker B, Walker S. Fitting linear mixed-effects models using lme4. J Stat Softw 2015;67:1–48. doi:10.18637/jss.v067.i01.

[6] Besag J, York J, Mollié A. Bayesian image restoration, with two applications in spatial statistics. Ann Inst Stat Math 1991;43:1–20.

[7] Rue H, Held L. Gaussian Markov random fields: theory and applications. CRC press; 2005.

[8] Rue H, Martino S, Chopin N. Approximate Bayesian inference for latent Gaussian models by using integrated nested Laplace approximations. J R Stat Soc Ser B Stat Methodol 2009;71:319–92. doi:10.1111/j.1467-9868.2008.00700.x.

[9] Martins TG, Simpson D, Lindgren F, Rue H. Bayesian computing with INLA: New features. Comput Stat Data Anal 2013;67:68–83. doi:10.1016/j.csda.2013.04.014.
